# Towards discovery of novel scaffold with potent antiangiogenic activity; design, synthesis of pyridazine based compounds, impact of hinge interaction, and accessibility of their bioactive conformation on VEGFR-2 activities

**DOI:** 10.1080/14756366.2019.1651723

**Published:** 2019-09-06

**Authors:** Maiy Y. Jaballah, Rabah A. T. Serya, Nasser Saad, Sohair M. Khojah, Marawan Ahmed, Khaled Barakat, Khaled A. M. Abouzid

**Affiliations:** a Faculty of Pharmacy, Pharmaceutical Chemistry Department, Ain Shams University, Abbassia, Cairo, Egypt;; b Faculty of Pharmaceutical Sciences and Pharmaceutical Industries, Pharmaceutical Chemistry Department, Future University in Egypt, Cairo, Egypt;; c Faculty of Science, Biochemistry Department, King Abdulaziz University, Jeddah, Kingdom of Saudi Arabia;; d Faculty of Pharmacy and Pharmaceutical Sciences, University of Alberta, Edmonton, AB, Canada;; e Li Ka Shing Institute of Virology, University of Alberta, Edmonton, AB, Canada;; f Li Ka Shing Applied Virology Institute, University of Alberta, Edmonton, AB, Canada;; g Faculty of Pharmacy, Department of Organic and Medicinal Chemistry, University of Sadat City, Menoufia, Egypt

**Keywords:** Pyridazine derivatives, VEGFR-2 inhibitors, antitumor agents, HUVEC, antiangiogenic agents, hinge interaction

## Abstract

Pyridazine scaffolds are considered privileged structures pertaining to its novelty, chemical stability, and synthetic feasibility. In our quest towards the development of novel scaffolds for effective vascular endothelial growth 2 (VEGFR-2) inhibition with antiangiogenic activity, four novel series of pyridazines were designed and synthesised. Five of the synthesised compounds; namely (**8c, 8f, 15, 18b, and 18c)** exhibited potent VEGFR-2 inhibitory potency (>80%); with IC_50_ values ranging from low micromolar to nanomolar range; namely compounds **8c, 8f, 15, 18c** with (1.8 µM, 1.3 µM, 1.4 µM, 107 nM), respectively. Moreover, 3-[4-{(6-oxo-1,6-dihydropyridazin-3-yl)oxy}phenyl]urea derivative **(18b)** exhibited nanomolar potency towards VEGFR-2 (60.7 nM). In cellular assay, the above compounds showed excellent inhibition of VEGF-stimulated proliferation of human umbilical vein endothelial cells at 10 μM concentration. Finally, an extensive molecular simulation study was performed to investigate the probable interaction with VEGFR-2.

## 1. Introduction

Angiogenesis is the process of sprouting or splitting of novel capillary blood vessels from the quiescent pre-existing vasculature^1^. Angiogenesis is normally orchestrated via a finely balanced equilibrium between pro-angiogenic and anti-angiogenic factors. However, aberrant equilibrium between these factors is associated with several human disorders, like rheumatoid arthritis, psoriasis, and cancer^2^.

The vascular endothelial growth 2 (VEGFR-2), also known as (KDR) has spurted as an attractive pharmacological target in cancer therapy pertaining to its crucial rule in tumorangiogenesis. Since the binding of VEGF to VEGFR-2 leads to receptor dimerisation, followed by the autophosphorylation of tyrosine residues in the intracellular kinase domain, resulting in potent mitogenic and chemotactic effects on endothelial cells (ECs)^3^.

The expression of VEGF is up-regulated by tumour-related changes, such as hypoxia, protooncogene activation, and the aberration of tumour-suppressor genes. Since the overexpression of VEGF correlates with poor prognosis and the clinical stage of patients with solid tumours, VEGF/VEGFR-2 signalling is thought to be an attractive target for the treatment of cancer.

Several VEGFR humanised anti-VEGF monoclonal antibody (bevacizumab) and small-molecule VEGFR-2 kinase inhibitors (Sorafenib^4^^,^^5^ Sunitinib^6^^,^^7^, and Pazopanib^8^) have been approved, and these have demonstrated clinical benefits in the treatment of some tumours with manageable side effects. Many angiogenesis inhibitors are also being evaluated in clinical trials for the treatment of various cancers.

**Figure 1.**
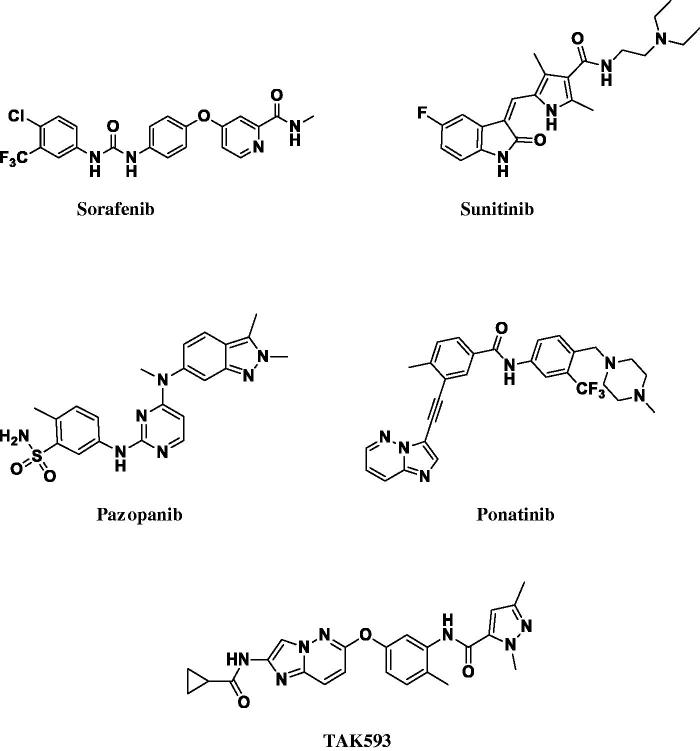
Structures of VEGFR-2 inhibitors approved for clinical use.

In the last two decade, pyridazine based scaffold has emerged as a novel, promising scaffold for the design and development of potent protein kinase inhibitors. Including either substituted pyridazine ring; or those incorporated in bicyclic ring system; such as imidazopyridazines. An imidazopyridazine derivative; Ponatinib^9^; evolved as a potent orally active Pan-inhibitor of (BCR-ABL) kinase, has reached phase I clinical evaluation of patients with refractory CML and other hematologic malignancies^10^. Moreover, **TAK-593**; an imidazo[1,2-*b*]pyridazine scaffold was designed and synthesised as a hinge binder^11^^,^^12^. It inhibits VEGFR1/2/3 (3.2/0.95/1.1 nM) and PDGFRα/β (4.3/13 nM). Oral administration of TAK-593 is currently being tested in a Phase I clinical trial in non-hematologic advanced cancer^13^ (Figure 1).

Despite the various advantages of pyridazines in drug design; including modulation of the physico-chemical properties, relatively ADME and toxicity profile, easy and diverse synthetic methods of access, as well as their affinity for a great number of receptor proteins; the potential inhibitory activity of pyridazine derivatives against VEGFR-2 is yet unravelled. Therefore, pyridazine derivatives were designed and synthesised in an attempt to elaborate a novel chemical scaffold for this purpose which could serve as alternative therapy for the current sorafenib and its analogues.

## 2. Rationale and design

**Figure 2.**
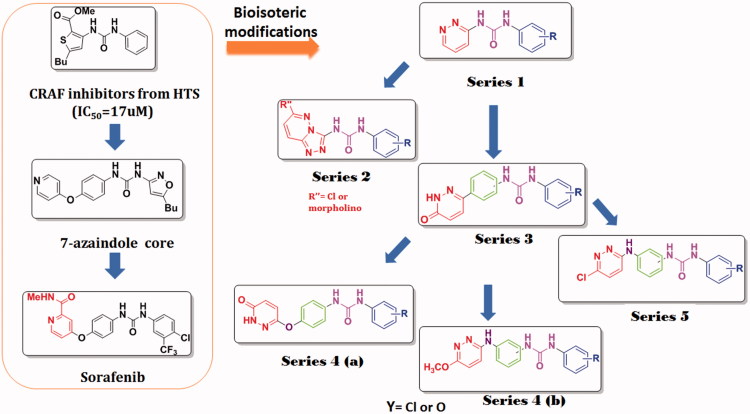
Design of the pyridazine- urea derivatives through scaffold hopping.

In light of the previous findings; we aimed at designing potent novel chemotypes as potent VEGFR-2 inhibitors. Thus, an array of pyridazine derivatives; including the unexplored triazolopyridazine derivatives through scaffold hopping strategy of the imidazopyridazine moieties were designed (Figure 2). Additionally, bioisosterism strategy was applied for designing pyridazine derivatives as VEGFR-2 inhibitors by replacing the central pyridine carboxamide core of sorafenib, a well-known VEGFR-2 inhibitor with IC_50_ value of 90 nM, with a pyridazine nucleus.

The newly synthesised pyridazine derivatives **3, 5a–c, 8a–f, 11a–f, 15, and 18a–c** were evaluated for their potential inhibitory activity toward VEGFR-2. Also, the most active derivatives were screened for their pendant antiangiogenic properties. Moreover, they were screened for their *in vitro* anticancer activity against a panel of 56 human cancer cell lines at NCI-USA.

## 3. Results and discussion

### 3.1. Chemistry

**Scheme 1.**
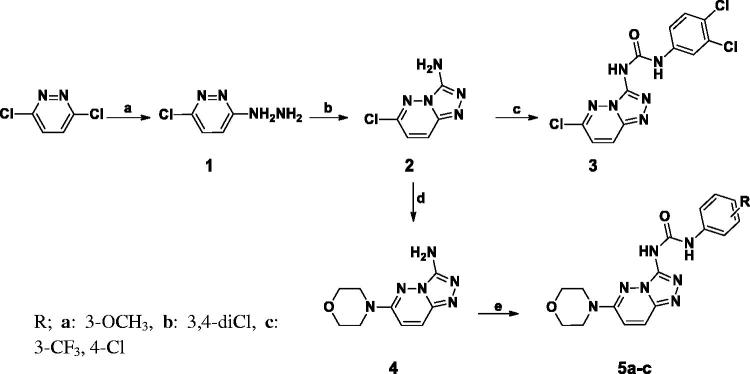
(a) NH_2_NH_2_.H_2_O (100%), ethanol, reflux, 3 h. (b) CNBr, ethanol, r.t. 24 h. (c) Substituted phenyl isocyanates, methylene chloride, TEA, r.t. 24 h. (d) Morpholine, *n*-butanol, reflux, 6 h. (e) Substituted phenyl isocyanates, dry THF, r.t. 24 h.

**Scheme 2.**
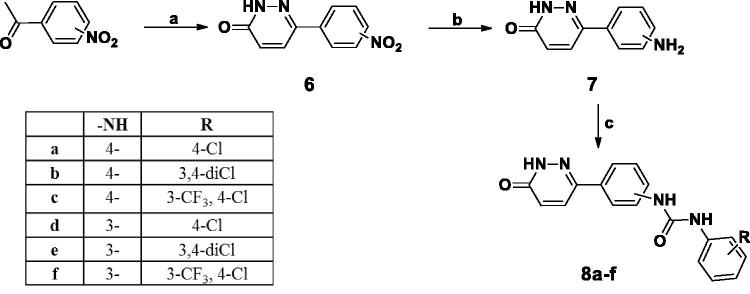
(a) i) Glyoxalic acid, glacial acetic acid, reflux, 48 h, then, ii) NH_2_NH_2_.H_2_O, reflux, 3 h, 45% (b) H_2_, Pd/C, Ethanol, 6 h. (c) Substituted phenyl isocyanates, dry THF, r.t. 24 h.

**Scheme 3.**
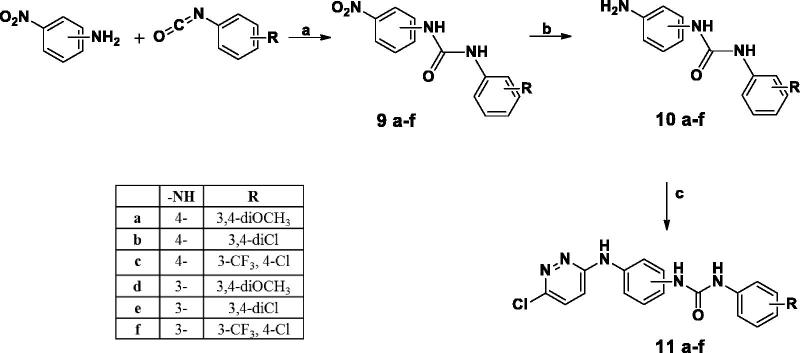
(a) Dry THF, rt, 24 h. (b) H_2_, Pd/C, EtOH, 120 min. (c) 3,6-dichloropyridazine, *n*-butanol, reflux, N_2_, 48 h, 35%

**Scheme 4.**
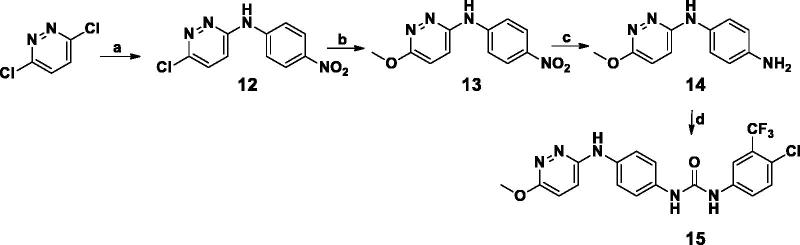
(a) 4-Nitroaniline, isopropanol, reflux, 6 h. (b) Sodium methoxide, DMF, reflux, 6 h. (c) H_2_, Pd/C, methanol, 6 h. (d) (3-trifloromethyl, 4- chloro phenyl ioscyanate), dry THF, r.t. 48 h.

**Scheme 5.**
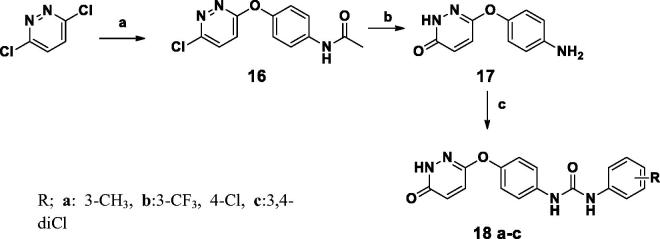
(a) Paracetamol, isopropanol, reflux, anhydrous K_2_CO_3_, 24 h, 75%. (b) i) Conc HCl, reflux, 1 h; ii) glacial acetic acid, reflux, 6 h. (c) Substituted phenyl isocyanates, dry THF, r.t. 24 h.

The synthetic strategies adopted for the preparation of the new pyridazines are described in Schemes 1–5. In Scheme 1, preparation of compound **2** was obtained through reaction of 3-chloro-6-hydrazinylpyridazine with cyanogen bromide. Which was either reacted with phenyl isocyanate derivative to furnish **3** or was reacted with morpholine, followed by the reaction with 3,4-dichloro phenyl isocyanates to afford compounds **5a–c**.

Scheme 2 describes the cyclo-condensation of either 4- or 3-nitroacetophenone derivatives to furnish the corresponding nitro phenyl pyridazinone derivatives (**6a,b**) respectively, which were later subjected to catalytic hydrogenation to afford the corresponding amines (**7a,b**) in high yield (90%) followed by nucleophilic substitution of substituted phenyl isocyanates to furnish phenyl pyridazinone urea derivatives **8a–f**.

On the other hand, in Scheme 3 the (6-chloropyridazin-3-yl)amino)phenyl derivatives were obtained via reflux the key intermediates **10a–f** with 3,6-dichloropyridazine in butanol under nitrogen atmosphere to afford compounds **11a–f.**

In Scheme 4, nucleophilic substitution of 3.6-dichloropyridaizine with 4-nitroaniline yielded 6-chloro-*N*-(4-nitrophenyl)pyridazin-3-amine; which was further reacted with sodium methoxide the 6-chloropyridazin-3-yl)nitro)phenyl derivatives was refluxed with sodium methoxide to furnish 6-methoxy-*N*-(4-nitrophenyl)pyridazin-3-amine (**14**) in high yield. Following catalytic hydrogenation of Compound (**14**); condensation with substituted phenyl isocyanate furnished compound **15**.

Scheme 5, describes the multistep synthesis of pendant compounds **18a–c**. Following nucleophilic substitution of 3,6-dichloropyridazine with paracetamol, the furnished compound was deacetylated following reflux in concentrated hydrochloric acid. The resulting 3-chloropyridazine derivative was then refluxed in glacial acetic acid to produce the 3-pyridazinone derivative (**17**) in excellent yield (85%). Following condensation with phenyl isocyanate derivatives, compounds **18a–c** were obtained in good yields (60–75%). The proposed structures of all newly prepared compounds were confirmed with spectral and elemental analyses.

### 3.2. Biological evaluations

#### 3.2.1. VEGFR-2 kinase inhibition assay

Initial screening at single dose of 10 µM concentration:

All the newly prepared compounds (***3, 5a–c, 8a–f, 11a–f, 15,* and *18a–c***) were tested *in vitro* for their kinase inhibitory activity against VEGFR-2. The percent inhibition of the enzymatic activity caused by the tested compounds against VEGFR-2 kinases was evaluated compared to reference kinase inhibitor staurosporine at a single concentration of 10 µM. The tested compounds depicted weak to excellent inhibitory activity against VEGFR-2 kinases (Table 1).

**Table 1.** VEGFR-2% inhibition, and IC_50_ of test compounds.

Cpd no.	R1	R2	R3	X	-NH position	VEGFR-2% inhibition^a^	VEGFR-2IC_50_^a^
3	Cl	Cl	Cl	–	–	3.06 ± 0.45	–
5a	Morphilino	OCH_3_	H	–	–	5.07 ± 0.61	–
5b	Morphilino	Cl	Cl	–	–	7.07 ± 1.11	–
5c	Morphilino	CF_3_	Cl	–	–	13.10 ± 1.71	–
8a	–	H	Cl	–	4	22.20 ± 1.85	–
8b	–	Cl	Cl	–	4	35.06 ± 2.87	–
8c	–	CF_3_	Cl	–	4	100.0 ± 4.61	1.3 ± 0.14 μM
8d	–	H	Cl	–	3	21.97 ± 2.73	–
8e	–	Cl	Cl	–	3	27.03 ± 3.29	–
8f	–	CF_3_	Cl	–	3	89.03 ± 2.97	1.8 ± 0.16 μM
11a	Cl	OCH_3_	OCH_3_	NH	4	21.93 ± 2.42	–
11b	Cl	Cl	Cl	NH	4	29.93 ± 4.12	–
11c	Cl	CF_3_	Cl	NH	4	22.07 ± 3.47	–
11d	Cl	OCH_3_	OCH_3_	NH	3	11.93 ± 2.25	–
11e	Cl	Cl	Cl	NH	3	20.13 ± 2.08	–
11f	Cl	CF_3_	Cl	NH	3	27.1 ± 1.93	–
15	OCH_3_	CF_3_	Cl	NH	4	97.1 ± 4.13	1.4 ± 0.12 μM
18a	O	CH_3_	H	O	4	15.06 ± 1.89	–
18b	O	CF_3_	Cl	O	4	96.03 ± 3.85	60.7 ± 0.03 nM
18c	O	Cl	Cl	O	4	100.27 ± 4.11	107 ± 0.04 nM
Strausporine						90.47 ± 4.53	–
Sorafenib^4^							90 nM^b^

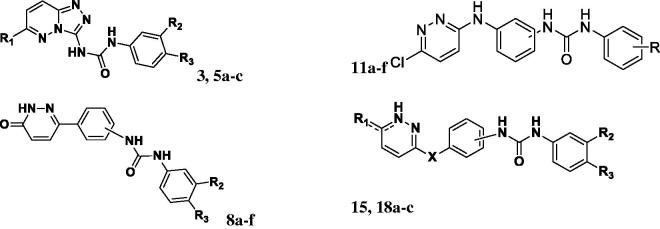

a Data are presented as Mean ± SD, *n* = 3.

b Reported IC_50_ values.

##### 3.2.1.1. SAR

The SAR study depends on the comparison between the inhibitory activity against VEGFR-2 and structural variations of pyridazine containing compounds.

Compounds in **series 1 (compounds 3, 5a–c)** exhibited weak inhibitory activity (3–7%) against VEGFR-2 at 10 µM concentration. Those compounds belong to the relatively short biaryl moieties. The rigidification of pyridazine containing compounds as well as substitution on the 6- position of the pyridazinyl moiety either with chloro or morpholino group (compounds **5a–c**) respectively did not improve the activity against VEGFR-2.

In **series 2**, (compounds **8a–f**) exhibited an improvement of activity was observed since two compounds **(8c, 8f)** of this series exhibited potent inhibition of VEGFR-2 kinase activity (100% and 89%, respectively). It is well-observed that incorporation of a central phenyl ring; directly attached to the pyridazine ring at the 3-position; as well as substitution of 6-position of the pyridazine moiety with oxygen atom, the activity markedly increased upon substituting the terminal aromatic side chain (3-trifloromethyl, 4-chloro) regardless the position of the urea linker (3- or 4-position as exhibited in compounds **8c** and **8f**, respectively). Interestingly, only (3-trifloromethyl, 4-chloro) substitution in the terminal phenyl ring is well-tolerated; whilst other substitutions (e.g. 3,4-di chloro in **8b**) had weak inhibition activity on VEGFR2 enzyme.

In **series 3**, further extension of the molecule via incorporation of extra linker NH linker **(compounds 11a–f)** did not improve the VEGFR inhibitory activity; since the compounds exhibited weak to moderate inhibition of the activity; ranging from (10–35%); whilst replacement of chlorine atom in the 6-position of the pyridazine ring in the above series with methoxy moiety (as in compound **15)** results in marked increase in the potency (89%).

Replacement of the 6-chloropyridazine with pyridazinone moiety; as well as incorporation of an ether linkage (as observed in compounds **18b,c)** lead to tremendous increase in the percentage inhibition (96% and 98%, respectively); provided that the terminal phenyl ring is di-substituted in the 3- and 4-positions (3, 4-dichloro; 3-trifloromethyl,4-chloro) substitution in **18b** and **18c**, respectively. Mono substitution results in a significant drop in activity, i.e. (**18a** with percentage inhibition of 32%).

##### 3.2.1.2. Measurement of potential enzyme inhibitory activity (IC_50_)

The tested compounds (**8c, 8f, 15, 18b, and 18c),** which displayed VEGFR-2 inhibition percent above 75% at 10 µM concentration were further investigated for their dose-related VEGFR-2 enzymatic inhibition at 5 different concentrations (1 nM - 10 nM - 100 nM - 1 µM - 10 µM) to subsequently calculate their IC*_50_* values (Table 1). Compounds **18b** and **18c**, potently inhibited VEGFR-2 at nanomolar IC_50_ values (60.7 ± 0.03 and 107 ± 0.04 nM, respectively). Also, compounds **8c**, **8f**, and **15** moderately inhibited VEGFR-2 with IC50 values of, 1.3 ± 0.14, 1.8 ± 0.16, and 1.4 ± 0.12 μM, respectively. Figures representing IC_50_ are provided in Supporting Information (Supplemental Figure S1)

These impressive results encouraged us to pursue further investigations regarding the activity of these compounds (Table 1).

#### 3.2.2. *In vitro* human umbilical vein endothelial cells (HUVEC) anti-proliferative assay

To Further investigate the potential anti-angiogenic properties of the investigated compounds; compounds that showed potent IC_50_ values against VEGFR-2; where subjected to HUVEC cell line anti-proliferative assay.

Angiogenesis process involves EC sprouting from the parent vessel, followed by migration, proliferation, alignment, tube formation, and anastomosis to other vessels. Several *in vitro* models have been attempted to recreate this complex sequence of events^14^. HUVECs have played a major role as a model system for the study of the regulation of EC function and the role of the endothelium in the response of the blood vessel wall to stretch, shear forces, and the development of atherosclerotic plaques and angiogenesis

Compounds **8c, 8f, 15, 18b, and 18c** were selected to be tested for their ability to *in vitro* inhibit VEGF-induced HUVEC cell line proliferation, using doxorubicin as a control.

The given test compounds manifested potent anti-proliferative activities against HUVEC cell line. Compound **(8f)** (VEGFR-2 IC*_50_* = 1827 nM) showed the highest growth inhibition (GI) percent (99.82%). The rest of compounds manifested moderate to high inhibition percent.

**Figure 3.**
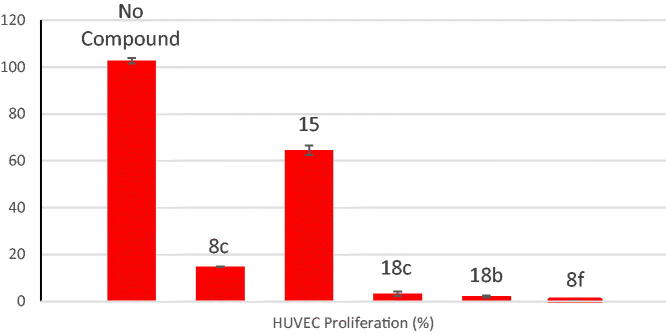
% HUVEC cell growth following treatment with compounds **8c, 8f, 15, 18b,** and **18c** compared to untreated control.

However, despite their potent VEGFR-2 inhibitory activity, compounds **(15)** exhibited moderate anti-proliferative activity against HUVEC cell line (35.5%; Table 2; Figure 3).

**Table 2.** Anti-proliferative activity against HUVEC cell line.

Compound (10 µM)	HUVEC proliferation (%)
No compound	102.58 ± 1.158
**8c**	14.75 ± 0.059
**8f**	0.18 ± 0.019
**15**	64.50 ± 2.017
**18c**	3.25 ± 0.435
**18b**	2.13 ± 0.425

Data are presented as Mean ± SD, *n* = 3.

#### 3.2.3. *In vitro* anti-cancer activity

The structures of the all synthesised compounds (**3, 5a–c,8a–f, 11a–f, 15, and 18a–c**) were submitted to the National Cancer Institute (NCI) Developmental Therapeutic Program (www.dtp.nci.nih.gov).

Seven compounds were selected to be screened for their anticancer activity *in vitro*. The anticancer assays were performed in accordance with the protocol of the Drug Evaluation Branch, NCI, Bethesda^15–17^.

The selected compounds were evaluated at primary anticancer assay against a panel of 56 cancer lines at concentration 10–5 M. The human tumour cell lines were derived from nine different cancer types: leukaemia, melanoma, lung, colon, CNS, ovarian, renal, prostate, and breast cancers.

**Figure 4.**
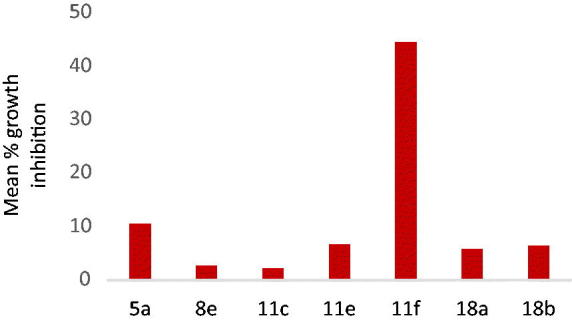
Mean % GIs of the tested compounds over NCI 56 cell line panel.

A 48 h drug exposure protocol was used and sulforhodamine B (SRB) protein assay was applied to estimate the cell viability and growth^18^. The mean percentages GI (GI%) of the tested compounds over the full panel of cell lines are illustrated in Figure 4.

**Figure 5.**
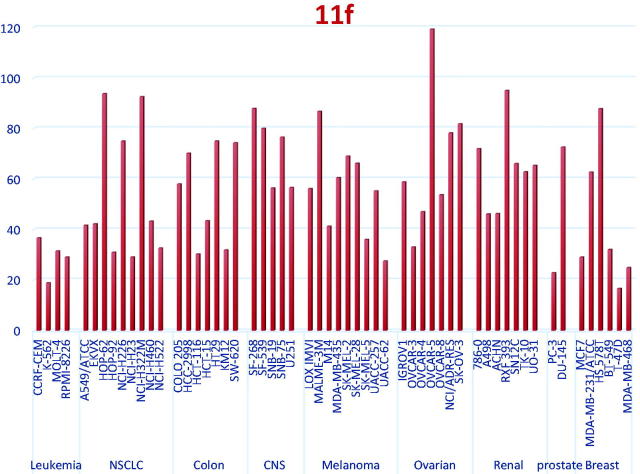
% Growth exerted by compound **11f** at 10 μM concentration over NCI 56 cell line panel.

The obtained data revealed that, the tested compounds from series 1 and 2 displayed fair anti-cancer activity (mean % GI = 11–24) except for compound **11f** featuring a lipophilic 3-trifloromethyl 4-chloro on the pendant phenyl which elicited the highest activity (mean % GI = 45). It exhibited broad spectrum and potent anti-proliferative activity against several NCI cell panel, namely; the leukaemic HL-60 (TB), MOLT-4, and SR cancer cell lines with GI 81.7%, 70.1%, and 81.2%, respectively, the non-small cell lung cancer A549/ATCC, NCI-H226, and NCI-H322M cell lines with cell GI 58.4%, 69.1%, and 71%, respectively, the colon cancer HCT-15, SW-620 cell line with 69.8% and 68.2% inhibition, respectively, the melanoma SK-MEL-5 and UACC-62 cell line with 64.72% inhibition, respectively. The ovarian cancer OVCAR-3 with 67% and the prostate cancer PC-3 cell line with 77.2%. Finally, it showed broad spectrum cell GI against the breast cancer MCF7, BT-549, T47D, and MDA-MB-468 with 71%, 68%, 83.4%, and 70.1%, respectively (Figure 5).

NCI selected compounds belonging to **Series 4**; i.e. pyridazin-6-one series linked to biarylureas via an ether linkage **(18b,c)**, both exhibited weak inhibitory activity against most of the test cell lines. The weak inhibitory activity of compound (**18b**) further confirms the selectivity against VEGFR-2 enzyme.

##### 3.2.3.1. Molecular modelling studies

**Figure 6.**
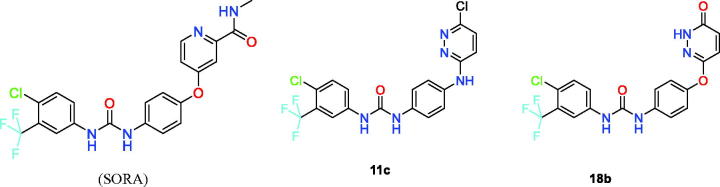
The chemical structures for the three compounds under the in-depth structural investigation, SORA, 11c, and 18b. SORA and 18b possess an ether linker whereas 11c has an amine linker. The torsional space around these linkers was scanned at the B3LYP/6–31 + G* level of theory.

As discussed in the methods section, three ligands were selected to perform the extensive *in silico* analysis. These ligands are, Sorafenib (**SORA**), **11c**, and **18b**. As shown in Figure 6, the 4-chloro-3-(trifluoromethyl)phenyl urea moiety is common between all ligands. The major structural difference between the three ligands is as follows. While SORA and **18b** are O-linked (ether linker) to the terminal hetero-aromatic ring, compound **11c** possesses an N-linker (amine linker). In the following sections, we will discuss how the nature of this linker affected the predicted binding mode of the three compounds and how this was reflected on the measured inhibitory effects of the compounds (Figure 6).

### 3.3. Detailed analysis of the potential binding modes of Sorafenib, 11c and 18b; analysing the binding modes and the simulation trajectories

**Figure 7.**
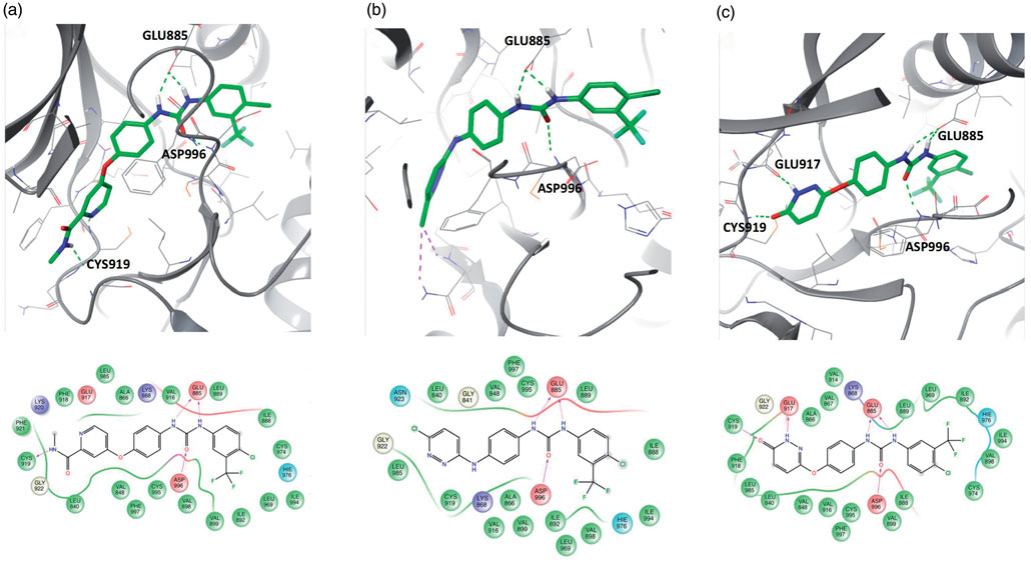
The ligand interaction diagrams of the compounds under study. The upper panel shows the three-dimensional binding modes whereas the lower panel shows the 2D diagrams of (a) Sorafenib, (b) **11c** and (c) **18b**.

The synthesised molecules were tested in enzymatic inhibition assay against VEGFR-2, in comparison to the positive control Strausporine. As shown in Table 1, compound **18b** shows the best enzymatic inhibition activity with an IC_50_ value of 60.7 nM. This was the reason for selecting this compound to perform the in-depth structural investigation. To avoid redundancy with the already discussed SAR analysis, we will limit the discussion here to the predicted binding modes of compounds **11c** and **18b** compared to SORA. The predicted binding modes of **SORA**, **11c,** and **18b** are shown in Figure 7. As shown in the Figure 7(a,c), SORA and **18b** are capable of forming strong H-bond with hinge residues, CYS919, and GLU917. On the other hand, the predicted binding conformation of compound **11c** lacks this H-bond as shown in Figure 7(b). As discussed in many studies, the formation of this specific H-bond is crucial for the activity of all reported competitive kinase inhibitors^19–22^. Interestingly, both SORA and compound **18b** form dual H-bonds at this region, which offers a significant boost to their affinity against VEGFR-2. Whereas, SORA forms these two H-bonds with CYS919, **18b** engages with GLU917 backbone amide bond in addition to CYS919. Our hydrogen bond occupancy analysis for the hinge H-bond (H-bond criteria 3.2 Å for the distance and 120° for the angle) with CYS919 showed conservation for this H-bond for approximately 86% of the simulation time in VEGFR-2 complex with SORA, and 98.4% for compound **18b**.

The second half of the three molecules, the 4-chloro-3-(trifluoromethyl)phenylcarbamoylamino moiety forms an optimal interaction with several residues at the VEGFR-2 pocket. As shown in Figure 7, the urea moiety forms two strong H-bonds with GLU885 and ASP996. The 4-chloro-3-(trifluoromethyl)phenyl substitution occupies the allosteric hydrophobic pocket of VEGFR-2. The presence of this pocket is a signature for the majority of type-II kinase inhibitors^23–25^; the DFG-out kinase inhibitors. The pocket is predominantly lipophilic, and thus only lipophilic substitution at this site region of the molecule can show stable binding. As also shown in Figure 7, residues ILE994, VAL898, ILE892, LEU969, ILE888, and LEU898 form the pocket.

### 3.4. The potential energy surface (PES) scan for the compounds under investigation; the impact of the ether/amine linker on the preferred bioactive conformation

For typical molecular docking software, two steps are necessary, which include a conformational sampling step followed by a scoring of the predicted binding poses. In general, an internal library of torsional angles can ensure that the conformational sampling algorithm generate energetically favourable ligand conformations based on its internal potential energy function^26^. In an ideal scenario, the sampled conformations of the small molecule will correspond to a minima on its PES, but not necessarily the global minimum. Theoretically, the introduction of additional interactions of the small molecule with the binding site residues, or any crystal contacts in general, should overcome small energy barriers against a less preferred conformation for this small molecule. However, a recent rigorous analysis report by Zheng et al has attempted to determine the conformational variations between gas phase and crystal conformers for 452 molecules^27^. Interestingly, the report found that in 50% of the cases, the crystal conformer lies within < 0.6 kcal/mol energy difference from the gas phase conformer. The study also found that it is only 10% of the cases the crystallised state of the molecule can adopt high-energy conformational structures, and this was mostly for highly polar compounds, such as sugars. Furthermore, recent SAR analysis studies from several research groups indicated that there was a strong correlation between the biological activity of the small molecule ligands and the relative abundance of the bioactive (bound-like) conformation in the free state^28^. These observations suggest that the critical examination of the proposed binding modes and the relative abundance of ligand bioactive conformation are essential parts of any meaningful SAR analysis.

**Figure 8.**
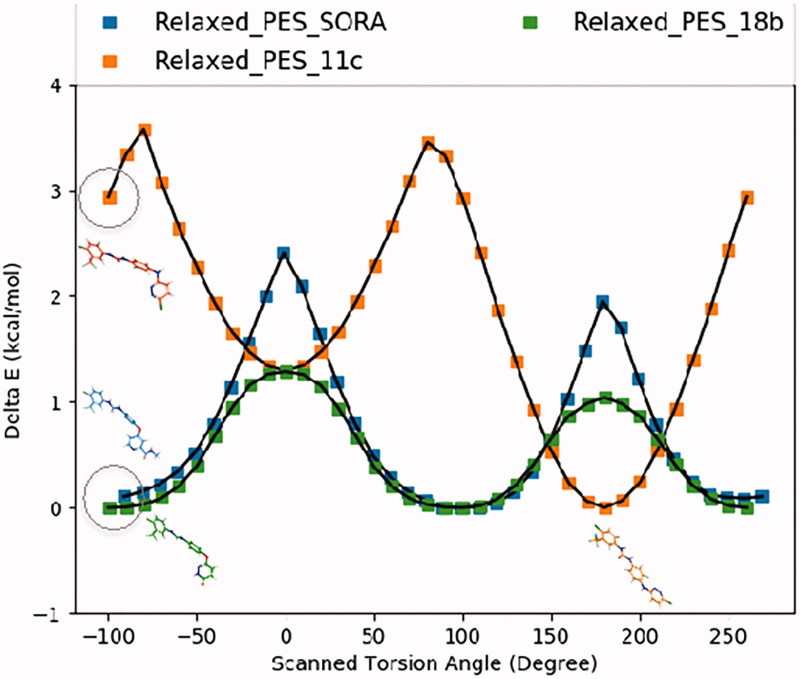
The relaxed PES scan of the torsional angel of the selected linker. The scan was initiated from a sorafenib bound-like conformation for all ligands such that they start and end approximately at the same value of the torsional angle. Representative conformations for the most stable (global energy minimum) conformation are given in the figure. For SORA and **18b**, this conformation corresponds to the bioactive conformation, whereas for **11c**, this conformation (planar) is not the bioactive one, i e, not the conformation required to form an H-bond with the hinge residues. The carbon atoms of each ligand are coloured similarly to the scanning steps. The scan was performed at the B3LYP/6–31 + G* level of theory.

In the current study, we were interested to investigate whether the nature of the linker between the two parts of the molecules, the 4-chloro-3-(trifluoromethyl)phenylurea moiety and the terminal hetero-aromatic ring system has an impact on the observed activity. Therefore, we conducted a relaxed PES scan for the torsion around this linker. The PES scan was conducted at the B3LYP/6–31 + G* level of theory via rotating the X-C torsional by 10° increment (X is an oxygen in SORA and **18b**, but a nitrogen in **11c**). The PES scans were performed for 36 steps to cover the full torsional space around this bond. We started the scan from a SORA like bound conformations for the three molecules, SORA, **11c**, and **18b**. Upon completion, the three PES scans were plotted together taken the global energy conformation of each molecule as a reference (0 kcal/mol) for its own PES. The PES scans of the three molecules are given in Figure 8.

As illustrated in the PES scans in Figure 8, the minimum energy conformers of compounds **18b** and SORA correspond to the bioactive, bound like conformations of these two molecules. The PES scan also indicated that the conformational preferences of SORA and compound **18b** are very similar. The ether linker on these two molecules favours a perpendicular (non-coplanar) orientation of the terminal hetero-aromatic ring with respect to the phenyl-carbamoylamino moiety. This perpendicular conformation corresponds to a global energy minimum on the PES surfaces of these two molecules. For compound **11c**, however, the planar orientation of the two terminal rings is favoured by approximately 3 kcal/mol from the perpendicular, SORA like conformation of compound **11c**. Our attempt to re-optimise the geometry the perpendicular conformation of compound **11c** at the M062X/6–311++G** level of theory again generated the planar conformation as the most stable geometry of this molecule. This data clearly indicates that the planar conformation of compound **11c** is much more favourable than the perpendicular conformation. Taken all the interactions that compound **11c** should form with the VEGFR-2 binding site residues, such as hinge, GLU885 and ASP996 as well as the lipophilic interaction with the allosteric pocket, only the perpendicular conformation of compound **11c** could potentially form these interactions. As this conformation is very unlikely to happen, given the high strain energy penalty, compound **11c** is expected to exhibit a very weak inhibition of VEGFR-2 (23% at 10 μM, see Table 1). We also expanded our analysis to compound **15** (amine linker) which has a methoxy substituted pyridazine ring and it was clear from the PES scan that a SORA like conformation is indeed favourable, only 1 kcal/mol higher energy than the global energy minimum structure. This may explain the good activity of compound **15** (IC_50_ = 1406 nm, %inhibition 98% at 10 μM) compared to compound **11c** (%inhibition 23% at 10 μM) in which the proposed bioactive conformation is 3 kcal/mol higher energy than the global energy conformer. The PES scan of **15** is given in Supporting Information (Supplemental Figure S2).

### 3.5. Molecular mechanics (MM)-GBSA binding energy and per-residue binding energy decomposition

Analysing the generated MD trajectories of the three ligands under investigation showed that the bound conformations of SORA and compound **18b** exhibited a much more stable trajectory over the simulation time (25 ns). The observed average ligand RMSD values for all ligands are within 1 Å, with compound **11c** being the highest (∼0.99 Å), that is slightly higher than SORA (∼0.64 Å) and 18 b (∼0.69 Å). The maximum ligand RMSD values are also exhibited by compound **11c** (∼1.54 Å), compared to SORA (∼1.39 Å) and compound **18b** (∼1.29 Å). The average receptor RMSD values for all ligands lie within ∼1.5 Å, and the maximum receptor RMSD value is below 2.5 Å for all trajectories. Ligand and receptors RMSD figures are given in the Supporting Information (Supplemental Figure S3).

The predicted binding affinities of the molecules have been estimated using the AMBER/MM-GBSA method as implemented in AMBER14. The numerical values are listed in Table 2, together with the predicted docking scores and H-bond occupancies over the generated trajectories. As shown in Supplemental Table S1, the predicted AMBER/MM-GBSA free energy of binding is nicely correlating with the measured inhibitory activities of the compounds. The AMBER/MM-GBSA free binding energy of SORA is given by –46.87 kcal/mol, approximately 3.49 kcal/mol better than that of compound **18b** (–43.38 kcal/mol) and 9.90 kcal/mol better than compound **11c** (–36.97 kcal/mol). The table also demonstrated the enhanced contribution of the AMBER/MM-GBSA lipophilic van der Waals (vdW) interaction (Δ*G*_MM-GBSA/vdW_) for all ligands compared to the electrostatic contributions (Δ*G*_MM-GBSA/ELE_) to the free binding energy. For example, the Δ*G*_MM-GBSA/vdW_ of SORA is –59.72 kcal/mol, –51.61 kcal/mol for compound **18b**, and –51.84 kcal/mol for compound **11c**. The enhanced lipophilic interaction of SORA compared to compounds **18b** and **11c** is arguably due to the presence of terminal methyl amino group that can form additional interaction with PHE918 and LEU840 residues at the ATP site. The lipophilic energy decomposition for the favourable residues is given in Supporting Information Supplemental Figure (S4). The Δ*G*_MM-GBSA/ELE_ electrostatics interaction term showed consistencies with the reported H-bond occupancies. As shown in Supplemental Table S1, the Δ*G*_MM-GBSA/ELE term_ for compound **18b** is –39.98 kcal/mol, slightly better than SORA (–37.95 kcal/mol), and significantly better than 11c (–29.12). The predicted docking scores listed in Supporting Information Supplemental Table S1 also show a similar trend with the AMBER/MM-GBSA, however, the docking scores strongly favour compound **11c**, which emphasise the importance of post processing of the docking results as mandatory step to achieve better performance.

## 4. Experimental

### 4.1. Chemistry

Melting points were measured with a Stuart melting point apparatus and were uncorrected. Infrared spectra were recorded as potassium bromide discs on Schimadzu FT-IR 8400Sspectrophotometer and expressed in wave number (cm^–1^). The NMR spectra were recorded by Varian Gemini-300BB at 300 MHz (Varian Inc., Palo Alto, CA) or Bruker spectrophotometer at 400 MHz. 1H NMR spectra were run at 300 or 400 MHz, while 13 C NMR spectra were run at 75or 100 MHz in deuterated dimethyl sulfoxide (DMSO-d6) or deuterated chloroform (CDCl3).

Chemical shifts (*δ*H) are reported relative to TMS as internal standard. All coupling constant (*J*) values are given in hertz. Chemical shifts (*δ*C) are reported relative to DMSO-*d6* as internal standard. The abbreviations used are as follows: *s*, singlet; *d*, doublet; *m*, multiplet. Mass spectra were measured on a GCMS-QP1000 EX and Helwett Packard 5988 spectrometers at 70 eV.

Elemental analyses were carried out at the Regional Center for Microbiology and Biotechnology, Al-Azhar University, Cairo, Egypt. Analytical thin layer chromatography on silica gel plates containing UV indicator was employed routinely to follow the course of reactions and to check the purity of products. All reagents and solvents were purified and dried by standard techniques.

#### 4.1.1. 3-Chloro-6-hydrazinylpyridazine (1)

Compound **1** was prepared according to reported procedure^29^ (m.p. 92 °C, as reported).

#### 4.1.2. 6-Chloro[1, 2, 4]triazolo[4,3-b]pyridazin-3-amine (2)

Compound **2** was prepared according to reported procedure^30^ (m.p. 287 °C as reported).

#### 4.1.3. 6-[1, 2, 4]Triazolo[4,3-b]pyridazin-3-amine (4)

Compound **4** was prepared according to reported procedure^29^ (m.p. 198–200 °C, as reported).

#### 4.1.4. 2–6-(4-Nitrophenyl)pyridazin-3(2H)-one628

Compounds **6** were prepared according to reported procedures^31^.

***6–(4-Aminophenyl)pyridazin-3(2H)-one (7)***^32^^,^^33^

Compounds **6** were prepared according to reported procedures^32^,

1–(3 or 4-Nitrophenyl)-3- substituted phenylurea **9a–f**

Compounds **9a–f** were prepared according to reported procedures^34–37^.

1–(3-Aminophenyl)-3-substituted phenyl urea (10a–f)

Compounds **10a–f** were prepared according to reported procedures^15^^,^^37–40^

***6-Chloro-N-(4-nitrophenyl)pyridazin-3-amine (12)***^16^

Compound **12** was prepared according to reported procedure^16^ (m.p. 182–183 °C as reported).

***6-Methoxy-N-(4-nitrophenyl)pyridazin-3-amine* (13)**^17^

Compound **13** was prepared according to reported procedure^17^ (m.p. 123–125 °C, as reported).

***N^1^-(6-methoxypyridazin-3-yl)benzene-1,4-diamine* (14)**

Compound **14** was prepared according to reported procedure^18^ (m.p. 132–134 °C, as reported).

***N*-(4-((6-Chloropyridazin-3-yl)oxy)phenyl)acetamide (16)**

Compound **16** was prepared according to reported procedure^41^^,^^42^ (m.p. 192–194 °C °C as reported)

***6–(4-Aminophenoxy)pyridazin-3(2H)-one (17)***

Compound **17** was prepared according to reported procedure^41^^,^^42^ (m.p. 130 °C as reported) General procedure for preparation of target compounds (**3, 5a–c, 8a–f, 11a–f, 15, 18a–c**)

To a solution of **the corresponding intermediate; compound 2, 4a–c, 7a–f, 10a–f, 14, and 17a–c, respectively** (6 mmol) in dry THF (20 ml), the appropriate isocyanate (6 mmol) was added and the mixture was stirred at room temperature for 24 h. The formed solid was collected by filtration, washed with minimal quantities of dry THF. Crystallisation was accomplished using appropriate solvent.

***1-[6-Chloro-[1, 2, 4]triazolo[4,3-b]pyridazin-3-yl)-3–(3,4-dichlorophenyl)]urea* (3)**

Acetonitrile giving compound **(XVII)** as white crystals (0.85 g, 40%); m.p. 173–175 °C; **^1^HNMR (300 MHz, DMSO-d_6_)** δ 10.00 (s, 1H, NH D_2_O exchangeable, NH urea), 9.66 (s, 1H, NH D_2_O exchangeable, NH urea), 8.12(s, 1H, ArH), 8.07 (d, *J* = 10.7 Hz, 1H, pyridazinyl H), 7.76 (d, *J* = 8.8 Hz, 1H, ArH), 7.64 (d, *J* = 8.8 Hz, 1H, ArH), 7.36 (d, *J* = 10.7 Hz, 1H, pyridazinyl H).**FT-IR (ύ max, cm^−1^)**: 3288–3272(NH), 3050 (CH aromatic), 2984 (CH aliphatic), 1685 (C = O amide), 1635 (C = N); **MS**: (Mwt.: 355.97) *m*/*z* (% rel. Int.) 357.01 (M^+^+1, 3.31), 355.99 (M^+^, 10.01), 176 (100); **Anal**. Calcd for C_12_H_7_Cl_3_N_6_O C, 40.31; H, 1.97; N, 23.50. Found: C, 40.42; H, 1.94; N, 23.63.

***1-[3,4-Dichlorophenyl)-3–(6-morpholino-[1, 2, 4]triazolo[4,3-b]pyridazin-3-yl]urea (5a)***

Crystallised from THF as yellowish white crystals (0.97 g, 40%); m.p. 145–147 °C; **^1^HNMR (300 MHz, DMSO-d_6_)** δ 10.00 (s, 1H, NH D_2_O exchangeable), 9.66 (s, 1H, NH D_2_O exchangeable), 8.12(s, 1H, ArH), 8.07 (d, *J* = 10.7 Hz, 1H), 7.76 (d, *J* = 8.8 Hz, 1H), 7.64 (d, *J* = 8.8 Hz, 1H), 7.36 (d, *J* = 10.7 Hz, 1H), 3.69 (d, *J* = 4.2 Hz, 4H, morpholinyl H), 3.53 (d, *J* = 4.2 Hz, 4H, morpholinyl H).

**^13^C NMR (DMSO-*d*6, 100 MHz)** d: 47.4 (2CH_2_ morpholine), 66.1 (2CH_2_ morpholine), 120.4, 124.0, 125.0, 126.3, 128.3, 128.9, 131.2, 131.9, 139.9, 141.7, 145.1, 155.4, 163

**FT-IR (ύ max, cm^−1^)**: 3291 (NH), 3122 (CH aromatic), 2988 (CH aliphatic), 1686 (C = O amide), 1640 (C = N); **MS**: (Mwt.: 408.24); **Anal**. Calcd for C_16_H_15_Cl_2_N_7_O_2_ C, 47.07; H, 3.70; N, 24.02. Found: C, 47.08; H, 3.73; N, 24.07.

***1-[4-Chloro-3-(trifluoromethyl)phenyl)-3–(6-morpholino-[1, 2, 4]triazolo[4,3-b]pyridazin-3-yl]urea* (5b)**

Crystallised from ethanol as buff crystals (1.19 g, 45%); m.p. 221–223 °C; **^1^HNMR (300 MHz, DMSO-d_6_)** δ 10.23 (s, 1H, NH D_2_O exchangeable), 9.67 (s, 1H, NH D_2_O exchangeable), 8.43(s, 1H, ArH), 8.22 (d, *J* = 9.9 Hz, 1H), 7.87 (d, *J* = 8.8 Hz, 1H), 7.64 (d, *J* = 8.8 Hz, 1H), 7.21 (d, *J* = 9.9 Hz, 1H), 3.73 (d, *J* = 4.2 Hz, 4H, morpholinyl H), 3.62 (d, *J* = 4.2 Hz, 4H, morpholinyl H).

**FT-IR (ύ max, cm^−1^)**: 3278–3288 (NH), 3112 (CH aromatic), 2990 (CH aliphatic), 1695 (C = O amide), 1636 (C = N); **MS**: (Mwt.: 441.78) *m*/*z* (% rel. Int.)443.82 (M^+^+2, 2.87), 442.82 (M^+^+1, 3.31), 441.75 (M^+^, 8.94), 151 (100); **Anal**. Calcd for C_17_H_15_ClF_3_N_7_O_2_ C, 46.22; H, 3.42; N, 22.19. Found: C, 46.27; H, 3.48; N, 22.22.

***1-[3-Methoxyphenyl)-3–(6-morpholino-[1, 2, 4]triazolo[4,3-b]pyridazin-3-yl]urea (5c)***

Crystallised from acetonitrile as yellowish white crystals (1.1 g, 50%); m.p. 189–192 °C; **^1^HNMR (300 MHz, DMSO-d_6_)** δ 9.87 (s, 1H, NH D_2_O exchangeable), 9.54 (s, 1H, NH D_2_O exchangeable), 8.45(s, 1H, ArH), 8.07 (d, *J* = 9.8 Hz, 1H), 7.82 (d, *J* = 8.2 Hz, 1H), 7.54 (d, *J* = 9.8 Hz, 1H), 7.36 (d, *J* = 8.2 Hz, 1H), 3.78 (d, *J* = 4.2 Hz, 4H, morpholinyl H), 3.69 (d, *J* = 4.2 Hz, 4H, morpholinyl H), 3.52(s, 3H, OCH_3_).

**FT-IR (ύ max, cm^−1^)**: 3301–3309 (NH), 3130 (CH aromatic), 2988 (CH aliphatic), 1686 (C = O amide), 1643 (C = N); **^13 ^C NMR (DMSO-*d*6, 100 MHz)** δ: 47.4 (2CH_2_ morpholine), 66.1 (2CH_2_ morpholine), 120.4, 124.0, 125.0, 126.3, 128.3, 128.9, 131.2, 131.9, 139.9, 141.7, 145.1, 155.4, 163. **MS**: (Mwt.: 369.38) *m*/*z* (% rel. Int.)371.42 (M^+^+2, 5.34), 270.39 (M^+^+1, 2.21), 368.38 (M^+^, 10.45), 152 (100); **Anal**. Calcd for C_17_H_19_N_7_O_3_ C, 55.28; H, 5.18; N, 26.54. Found: C, 55.32; H, 5.19; N, 26.64.

***1-[3,4-Dichlorophenyl)-3–(4-(6-oxo-1,6-dihydropyridazin-3-yl)phenyl)]urea* (8a)**

Crystallised from isopropanol as yellowish crystals(1.12g., 50%) m.p.: 232–235 °C. **^1^HNMR (300 MHz, DMSO-d_6_** δ 12.25 (s, 1H, D_2_O exchangeable, pyridazinyl NH), 9.19 (s, 1H, D_2_O exchangeable, NH urea), 8.93 (s, 1H,D_2_O exchangeable, NH urea), 8.14(s, 1H, ArH), 7.71 (*dd*, *J* = 8.8 Hz, 2H, ArH), 7.56(d, *J* = 9.9 Hz, 1H, pyridazinyl H), 7.49 – 7.30 (m, 2H, ArH), 7.13 (*dd*, *J* = 8.8 Hz, 2H, ArH), 6.99 (d, *J* = 9.9 Hz, 1H, pyridazinyl H).**MS**: (Mwt.: 374.03); **FT-IR (ύ max, cm^−1^)** 3286–3300 (NH), 3125 (CH aromatic), 1690 (C = O amide), 1640 (C = N); **Anal**. Calcd for C_17_H_12_Cl_2_N_4_O_2_: C, 54.42; H, 3.22; N, 14.93; Found: C, 54.65; H, 3.43; N, 14.98.

***1-[(4-Chloro-3-(trifluoromethyl)phenyl)-3–(4-(6-oxo-1,6-dihydropyridazin-3-yl)phenyl)]urea* (8b)**

Crystallised from THF as orange crystals (1.12 g., 50%) m.p.: 245–246 °C. **^1^HNMR (300 MHz, DMSO-d_6_** δ 12.35 (s, 1H,D_2_O exchangeable, pyridazinyl NH), 9.22 (s, 1H, D_2_O exchangeable, NH urea), 8.89 (s, 1H,D_2_O exchangeable, NH urea), 8.45(s, 1H, ArH), 7.54 (*dd*, *J* = 8.3 Hz, 2H, ArH), 7.32(d, *J* = 9 Hz, 1H, pyridazinyl H), 7.25 – 7.19 (m, 2H, ArH), 7.05 (*dd*, *J* = 8.3 Hz, 2H, ArH), 6.89(d, *J* = 9 Hz, 1H, pyridazinyl H).

**FT-IR (ύ max, cm^−1^)**: 3299–3310 (NH), 3038 (CH aromatic), 2915 (CH aliphatic), 1682 (C = O amide), 1633 (C = N); **MS**:(Mwt.: 408.76) *m*/*z* (% rel. Int.) 410.76 (M^+^+2, 4.08), 411.79 (M^+^+1, 3.31), 408.81(M^+^, 11.99), 194 (100); **Anal**. Calcd for C_18_H_12_ClF_3_N_4_O_2_: C, 52.89; H, 2.96; N, 13.71; Found: C, 52.72; H, 3.01; N, 13.85.

***1-[4–(6-Oxo-1,6-dihydropyridazin-3-yl)phenyl)-3–(3-(methoxy)phenyl)]urea* (8c)**

Crystallised from ethanol as buff crystals (1.35 g, 67%) m.p.: 201–203°. **NMR (300 MHz, DMSO-d_6_**δ 12.45 (s, 1H, D_2_O exchangeable, pyridazinyl NH), 9.21 (s, 1H, D_2_O exchangeable, NH urea), 8.89 (s, 1H, D_2_O exchangeable, NH urea), 7.46 (d, *J* = 8.2 Hz, 1H, pyridazinyl H), 7.37 (*dd*, *J* = 9.4 Hz, 2H, ArH), 7.25–7.09 (m, 2H, ArH), 6.99 (*dd*, *J* = 9.4 Hz, 2H, ArH), 6.87 (d, *J* = 8.2 Hz, 1H, pyridazinyl H), 3.52(s, 3H, OCH_3_). **^13^C NMR (DMSO-*d*6, 100 MHz)**δ:118.7, 119.7, 122.1, 125.1, 126.3, 127.4, 127.4, 130.4, 132.3, 132.6, 134.3, 137.2, 139.9, 140.1, 154.1,160.8. **FT-IR (ύ max, cm^−1^)**: 3293 (NH), 3089 (CH aromatic), 2954 (CH aliphatic), 1685 (C = O amide), 1638 (C = N); **MS**: (Mwt.: 336.34) **Anal**. Calcd for C_18_H_16_N_4_O_3_: C, 64.28; H, 4.79; N, 16.66. Found: C, 64.34; H, 4.92; N, 16.72.

***1-[3,4-Dichlorophenyl)-3–(3-(6-oxo-1,6-dihydropyridazin-3-yl)phenyl)]urea* (8d)**

Crystallised from methanol as white crystals(0.78g., 35%) m.p.: 232–233 °C. **^1^HNMR (300 MHz, DMSO-d_6_** δ 13 (s, 1H, D_2_O exchangeable, pyridazinyl NH), 9.19 (s, 1H, D_2_O exchangeable, NH urea), 8.93 (s, 1H, D_2_O exchangeable, NH urea), 8.01–7.92(m, 2H, ArH), 7.71 – 7.52 (m, 3H, ArH), 7.49 (s, *J* = 8.8 Hz, 1H, pyridazinyl H), 7.30 – 7.13 (m, 2H, ArH), 6.99 (d, *J* = 8.8 Hz, 1H, pyridazinyl H).

.**FT-IR (ύ max, cm^−1^)**: 3288–3297 (NH), 3118 (CH aromatic), 2994 (CH aliphatic), 1677 (C = O amide), 1631 (C = N); **MS**:(Mwt.: 374.21) *m*/*z* (% rel. Int.) 375.32 (M^+^+1, 5.42), 374.32 (M^+^, 8.94), 151 (100) **Anal**. Calcd for C_17_H_12_Cl_2_N_4_O_2_: C, 54.42; H, 3.22; N, 14.93. Found: C, 54.43; H, 3.27; N, 14.82.

***1-[4-Chloro-3-(trifluoromethyl)phenyl)-3–(3-(6-oxo-1,6-dihydropyridazin-3-yl)phenyl]urea* (8e)**

Crystallised from THF as yellowish crystals (1.02 g., 42%) m.p.: 198–200 °C. **^1^HNMR (300 MHz, DMSO-d_6_**δ 12.22 (s, 1H, D_2_O exchangeable, pyridazinyl NH), 8.99 (s, 1H, D_2_O exchangeable, NH urea), 8.54 (s, 1H, D_2_O exchangeable, NH urea),8.32 (s, 1H, ArH), 7.97 (d, *J* = 9.9 Hz, 1H, pyridazinyl H), 7.40 (m, 1H, ArH), 7.23 – 7.11 (m, 2H, ArH), 6.94 (m, 3H, ArH), 6.82 (d, *J* = 9.9 Hz, 1H, pyridazinyl H). **FT-IR (ύ max, cm^−1^)**: 3302 (NH), 3028 (CH aromatic), 2942 (CH aliphatic), 1690 (C = O amide), 1643 (C = N); **MS**: (Mwt.: 408.72) *m*/*z* (% rel. Int.) 410.72(M^+^+2, 6.23), 411.79, 408.81 (M^+^, 18.65), 194 (100); **Anal**. Calcd for C_18_H_13_F_3_N_4_O_2_C, 57.76; H, 3.50; N, 14.97. Found: C, 57.82; H, 3.62; N, 14.86.

***1-[(3-Methoxyphenyl)-3–(3-(6-oxo-1,6-dihydropyridazin-3-yl)phenyl)]urea* (8f)**

Crystallised from THF as pale yellow crystals(0.37g., 71%) m.p.: 221–223 °C. **^1^HNMR (300 MHz, DMSO-d_6_**δ 13.01 (s, 1H, D_2_O exchangeable, pyridazinyl NH), 8.89 (s, 1H, D_2_O exchangeable, NH urea), 8.67 (s, 1H, D_2_O exchangeable, NH urea), 7.99 (s, 1H, ArH), 7.86 (d, *J* = 9.3 Hz, 1H, pyridazinyl H), 7.45 – 7.23 (m, 3H, ArH), 7.08 (m, 3H, ArH), 6.92 (d, *J* = 9.3 Hz, 1H, pyridazinyl H), 3.63(s, 3H, OCH_3_) 13 C NMR (DMSO-d6, 100 MHz) δ:119.8, 120.4, 123, 124, 124.1, 125, 125.1, 126.3, 130.1, 131.2, 131.9, 134.3, 137.4, 139.7, 139.9, 143.9, 154.1, 160.8. **FT-IR (ύ max, cm^−1^)**: 3302 (NH), 3038 (CH aromatic), 2945 (CH aliphatic), 1682 (C = O amide), 1625 (C = N); **MS**: (Mwt.: 336.34); **Anal**. Calcd for C_18_H_16_N_4_O_3_ C, 64.28; H, 4.79; N, 16.66. Found: C, 64.42; H, 4.91; N, 16.72.

***1-[3-((6-Chloropyridazin-3-yl)amino)phenyl)-3–(3,4-dichlorophenyl)]urea* (11a)**

Crystallised from THF to give compound **(XXXa)** as white crystals (0.8 g, 20%); m.p. 238–240 °C; **^1^HNMR (300 MHz, DMSO-d_6_)** δ 9.98(s, 1H, NH D_2_O exchangeable, NH urea), 9.77(s, 1H, NH D_2_O exchangeable, NH urea), 8.99 (s, 1H, NH D_2_O exchangeable, NH Aryl), 8.74 (s, 1H, ArH), 7.84 (d, *J* = 9.3 Hz, 1H, pyridazinyl H), 7.61 – 7.45 (m, 2H, ArH), 7.38 (t, *J* = 6.4 Hz, 1H, ArH), 7.29 (d, *J* = 6.4 Hz, 1H, ArH), 7.18– 7.02 (m, 2H, ArH), 6.89 (d, *J* = 9.3 Hz, 1H, pyridazinyl H).

**FT-IR (ύ max, cm^−1^)**: 3347–3325 (NH), 3117 (CH aromatic), 2994 (CH aliphatic), 1682 (C = O amide), 1644 (C = N); **MS**: (Mwt.: 408.67) **Anal**. Calcd for C_17_H_12_Cl_3_N_5_O: C, 49.96; H, 2.96; N, 17.14. Found: C, 49.98; H, 2.87; N, 17.25.

***4-[Chloro-3-(trifluoromethyl)phenyl)-3–(3-((6-chloropyridazin-3-yl)amino)phenyl)]urea (11b)***

Crystallised from methanol to give compound **(XXXb)** as buff crystals (1.1 g, 25%); m.p. >250 °C; **^1^HNMR (300 MHz, DMSO-d_6_)** δ 10.12 (s, 1H, NH D_2_O exchangeable, NH urea), 9.86(s, 1H, NH D_2_O exchangeable, NH urea), 8.87 (s, 1H, NH D_2_O exchangeable, NH Aryl), 8.65 (s, 1H, ArH), 8.12 (d, *J* = 9 Hz, 1H, pyridazinyl H), 7.56 – 7.43 (m, 2H, ArH), 7.38 (d, *J* = 8.4 Hz, 1H, ArH), 7.29 (d, *J* = 8.4 Hz, 1H, ArH), 7.06– 6.99 (m, 2H, ArH), 6.9(d, *J* = 9 Hz, 1H, pyridazinyl H).

**FT-IR (ύ max, cm^−1^)**: 3338–3314 (NH), 3051 (CH aromatic), 2981 (CH aliphatic), 1664 (C = O amide), 1664 (C = N); **MS**: (Mwt.: 442.22); *m*/*z* (% rel. Int.) 444.24 (M^+^+2, 6.40), 442.35 (M^+^, 31.12), 168.27(100) **Anal**. Calcd for C_18_H_12_Cl_2_F_3_N_5_O: C, 48.89; H, 2.74; N, 15.84. Found: C, 48.92; H, 2.83; N, 15.92.

***1-[(3-((6-Chloropyridazin-3-yl)amino)phenyl)-3–(3,5-dimethoxyphenyl)]urea* (11c)**

Crystallised from methanol to give compound **(XXXc)** as yellowish white crystals (1.39 g, 35%); m.p. 195–197 °C; **^1^HNMR (300 MHz, DMSO-d_6_)** δ 10.23 (s, 1H, NH D_2_O exchangeable, NH urea), 9.76(s, 1H, NH D_2_O exchangeable, NH urea), 9.25 (s, 1H, NH D_2_O exchangeable, NH Aryl), 8.74 (s, 1H, ArH), 8.56 (d, *J* = 10.4 Hz, 1H, pyridazinyl H), 8.46 (d, *J* = 8.2 Hz, 1H, ArH), 8.25 – 8.03 (m, 2H, ArH), 7.89 (d, *J* = 8.2 Hz, 1H, ArH), 7.76– 7.53 (m, 2H, ArH), 7.24(d, *J* = 10.4 Hz, 1H, pyridazinyl H), 3.65(s, 3H, OCH_3_), 3.47(s, 3H, OCH_3_).

**FT-IR (ύ max, cm^−1^)**: 3347–3357 (NH), 3117 (CH aromatic), 2994 (CH aliphatic), 1682 (C = O amide), 1644 (C = N); **MS**:(Mwt.: 399.08):*m*/*z* (% rel. Int.) 401.11(M^+^+2, 1.39), 399.08(M^+^, 4.18), 219.12 (100); **Anal**. Calcd for C_19_H_18_ClN_5_O_3_: C, 57.07; H, 4.54; N, 17.52; Found: C, 57.12; H, 4.58; N, 17.59.

***1-[4-((6-Chloropyridazin-3-yl)amino)phenyl)-3–(3,4-dichlorophenyl]urea* (11d)**

Crystallised from ethanol(0.8 g, 20%); m.p. 252–254 °C; **^1^HNMR (300 MHz, DMSO-d_6_)** δ 10.02 (s, 1H, NH D_2_O exchangeable, NH urea), 9.64(s, 1H, NH D_2_O exchangeable, NH urea), 9.46 (s, 1H, NH D_2_O exchangeable, NH Aryl), 8.46 (s, 1H, ArH), 8.32 (d, *J* = 9.2 Hz, 1H, pyridazinyl H), 8.15 (*dd*, *J* = 8.8 Hz, 2H, ArH), 8.01–7.87 (m, 2H, ArH), 7.56 (*dd*, *J* = 8.8 Hz, 2H, ArH), 7.24(d, *J* = 9.2 Hz, 1H, pyridazinyl H).

**FT-IR (ύ max, cm^−1^)**: 3299–3312 (NH), 3040 (CH aromatic), 2980 (CH aliphatic), 1683 (C = O amide), 1638 (C = N); **MS**: (Mwt.: 408.67); **Anal**. Calcd for C_17_H_12_Cl_3_N_5_O: C, 49.96; H, 2.96; N, 17.14. Found. C, 49.99; H, 2.98; N, 17.16.

***1-[4-Chloro-3-(trifluoromethyl)phenyl)-3–(4-((6-chloropyridazin-3-yl)amino)phenyl)]urea* (11e)**

Crystallised from acetonitrile (1.1 g, 25%); m.p. 149–151 °C; **^1^HNMR (300 MHz, DMSO-d_6_)** δ 9.77(s, 1H, NH D_2_O exchangeable, NH urea), 9.54 (s, 1H, NH D_2_O exchangeable), 9.46 (s, 1H, NH D_2_O exchangeable, NH Aryl), 8.74(s, 1H, ArH), 8.15 (*dd*, *J* = 8.9 Hz, 2H, ArH), 7.84(d, *J* = 9.3 Hz, 1H, pyridazinyl H), 7.61 – 7.45 (m, 2H, ArH), 7.38(*dd*, *J* = 8.9 Hz, 2H, ArH), 7.12 (d, *J* = 9.3 Hz, 1H, pyridazinyl H).

**FT-IR (ύ max, cm^−1^)**: 3291 (NH), 3110 (CH aromatic), 2982 (CH aliphatic), 1690 (C = O amide), 1645 (C = N); **MS**:(Mwt.: 442.22); *m*/*z* (% rel. Int.) 444.34(M^+^+2, 4.32), 442.26(M^+^, 4.18), 168.56(100) **Anal**. Calcd for C_18_H_12_Cl_2_F_3_N_5_O: C, 48.89; H, 2.74; N, 15.84. Found: C, 48.95; H, 2.82; N, 15.89.

***1-[(4-((6-Chloropyridazin-3-yl)amino)phenyl)-3–(3,5-dimethoxyphenyl)]urea* (11f)**

Crystallised from methanol/water (0.59 g, 15%); m.p. 203–205 °C; **^1^HNMR (300 MHz, DMSO-d_6_)** δ 10.01(s, 1H, NH D_2_O exchangeable, NH urea), 9.77 (s, 1H, NH D_2_O exchangeable), 9.32 (s, 1H, NH D_2_O exchangeable, NH Aryl), 8.45(s, 1H, ArH), 8.32 (*dd*, *J* = 8.8 Hz, 2H, ArH),7.76(d, *J* = 9.3 Hz, 1H, pyridazinyl H), 7.54 – 7.32 (m, 2H, ArH), 7.21(*dd*, *J* = 8.8 Hz, 2H, ArH), 6.89 (d, *J* = 9.3 Hz, 1H, pyridazinyl H), 3.57(s, 3H, OCH_3_), 3.54(s, 3H, OCH_3_)

**FT-IR (ύ max, cm^−1^)**: 3310–3292 (NH), 3120 (CH aromatic), 2924 (CH aliphatic), 1690 (C = O amide), 1644 (C = N); **MS**:(Mwt.: 399.11) *m*/*z* (% rel. Int.) 401.16 (M^+^+2, 1.39), 399.09(M^+^, 4.18), 219.23 (100); **Anal**. Calcd for C_19_H_18_Cl_2_N_5_O_3_: C, 57.07; H, 4.54; N, 17.52. Found: C, 57.21; H, 4.59; N, 17.62

1–(4-Chloro-3-(trifluoromethyl)phenyl)-3–(4-((6-methoxypyridazin-3-yl)amino) phenyl) urea 15

Crystallised from isopropanol giving compound **XXXVII** (1.5 g, 60%); m.p. (>250 °C).

**^1^HNMR (300 MHz, DMSO-d_6_)** δ 9.97(s, 1H, NH D_2_O exchangeable, NH urea), 9.09(s, 1H, NH D_2_O exchangeable, NH urea), 8.95(s, 1H, NH D_2_O exchangeable, NH Aryl), 8.68(s, 1H, ArH), 8.11(d, *J* = 9.2 Hz, 1H, pyridazinyl H), 7.64(*dd*, *J* = 9.6 Hz, 2H, ArH), 7.37–7.19(m, 2H, ArH), 7.08 (*dd*, *J* = 9.6 Hz, 2H, ArH), 6.99 (d, *J* = 9.2 Hz, 1H, pyridazinyl H), 3.92 (s, 3H, OCH_3_).

**FT-IR (ύ max, cm^−1^)**: 3347 (NH), 3117 (CH aromatic), 2994 (CH aliphatic), 1682 (C = O amide), 1644 (C = N); (Mwt.: 437.80) *m*/*z* (% rel. Int.) 439.89 (M^+^+2, 10.64), 437.82(M^+^, 3.23), 366.10 (41.23), 215.09 (100).**Anal**. Calcd for C_19_H_15_ClF_3_N_5_O_2_ C, 52.12; H, 3.45; N, 16.00. Found: C, 52.34; H, 3.52; N, 16.07.

1–(3,4-Dichlorophenyl)-3–(4-((6-oxo-1,6-dihydropyridazin-3-yl)oxy)phenyl)urea 18a

Crystallised from methanol as yellowish white crystals (1.1 g, 50%); m.p. 189–190 °C; **^1^HNMR (300 MHz, DMSO-d_6_)** δ 12.25 (s, 1H, D_2_O exchangeable, pyridazinyl NH), 9.19 (s, 1H, D_2_O exchangeable, NH urea), 8.93 (s, 1H, D_2_O exchangeable, NH urea), 7.82 (d, *J* = 9.2 Hz, 1H, pyridazinyl H), 7.71–7.62 (m, 2H, ArH), 7.40 (*dd*, *J* = 8.3 Hz, 2H, ArH), 7.35(*dd*, *J* = 8.3 Hz, 2H, ArH), 7.23 – 7.11 (m, 1H, ArH), 6.82 (d, *J* = 9.9 Hz, 1H, pyridazinyl H).

**FT-IR (ύ max, cm^−1^)**: 3288–3296 (NH), 3047 (CH aromatic), 2990 (CH aliphatic), 1685 (C = O amide), 1632 (C = N); **MS**: (Mwt.: 391.21); **Anal**. Calcd for C_17_H_12_Cl_2_N_4_O_3_ C, 52.19; H, 3.09; N, 14.32. Found: C, 52.23; H, 3.17; N, 14.45.

1–(4-Chloro-3-(trifluoromethyl)phenyl)-3–(4-((6-oxo-1,6-dihydropyridazin-3-yl)oxy)phenyl)urea **18b**

Crystallised from ethanol as white crystals (1.1 g, 50%); m.p. 201–203 °C; **^1^HNMR (300 MHz, DMSO-d_6_)** δ 12.02 (s, 1H, D_2_O exchangeable, pyridazinyl NH), 9.45 (s, 1H, D_2_O exchangeable, NH urea), 8.72 (s, 1H, D_2_O exchangeable, NH urea), 8.32 (s, 1H, ArH), 8.02 (d, *J* = 9.2 Hz, 1H, pyridazinyl H), 7.30 (*dd*, *J* = 10.2, 8.3 Hz, 2H, ArH), 7.21(*dd*, *J* = 10.2, 8.3 Hz, 2H, ArH), 7.15 – 7.06 (m, 2H, ArH), 6.99 (d, *J* = 9.9 Hz, 1H, pyridazinyl H).

**FT-IR (ύ max, cm^−1^)**: 3285–3297 (NH), 3129 (CH aromatic), 2979 (CH aliphatic), 1692 (C = O amide), 1635 (C = N); **MS**: (Mwt.: 424.06) *m*/*z* (% rel. Int.) 426.06 (M^+^+2, 2.55), 427.11 (M^+^+1, 6.56), 424.06 (M^+^, 7.68), 198 (100); **Anal**. Calcd for C_20_H_18_ClF_3_N_4_O_3_ C, 50.90; H, 2.85; N, 13.19; Found: C, 50.98; H, 2.87; N, 13.20.

1–(3-Methoxyphenyl)-3–(4-((6-oxo-1,6-dihydropyridazin-3-yl)oxy)phenyl)urea 18c.

Crystallised from acetonitrile as yellowish white crystals (1.1 g, 50%); m.p. 156–157 °C; **^1^HNMR (300 MHz, DMSO-d_6_)** δ 12.05 (s, 1H, D_2_O exchangeable, pyridazinyl NH), 9.09 (s, 1H, D_2_O exchangeable, NH urea), 8.87 (s, 1H, D_2_O exchangeable, NH urea), 7.85 (d, *J* = 9.2 Hz, 1H, pyridazinyl H), 7.72–7. 65(m, 2H, ArH), 7.40 (*dd*, *J* = 8.3 Hz, 2H, ArH), 7.25(*dd*, *J* = 8.3 Hz, 2H, ArH), 7.13 – 7.01 (m, 1H, ArH), 6.22 (d, *J* = 9.2 Hz, 1H, pyridazinyl H).3.52(s, 3H, OCH_3_).

**FT-IR (ύ max, cm^−1^)**: 3314 (NH), 3141 (CH aromatic), 2981 (CH aliphatic), 1661 (C = O amide), 1661 (C = N); **MS**: (Mwt.: 352.12); **Anal**. Calcd for C_18_H_16_N_4_O_4_ C, 61.36; H, 4.58; N, 15.90. Found: C, 61.42; H, 4.61; N, 15.95.

### 4.2. Biological evaluation

#### 4.2.1. Measurement of inhibitory activity against VEGFR-2

The kinase activity of VEGFR-2 was measured by use of a phosphotyrosine antibody with the

Alpha Screen system (PerkinElmer, United States) according to manufacturer’s instructions. Enzyme reactions were performed in 50 mM Tris–HCl pH 7.5, 5 mM MnCl2, 5 mM MgCl2, 0.01% Tween-20 and 2 mM DTT, containing 10 μM ATP, 0.1 μg/mL biotinylated poly-GluTyr (4:1), and 0.1 nM of VEGFR-2 (Millipore, United Kingdom). Prior to catalytic initiation with ATP, the tested compounds at final concentrations ranging from 0 to 100 μg/mL and enzyme were incubated for 5 min at room temperature. The reactions were quenched by the addition of 25 μL of 100 mM EDTA, 10 μg/mLAlpha Screen streptavidine donor beads and 10 μg/mL acceptor beads in 62.5 mM HEPES pH 7.4, 250 mM NaCl, and 0.1% BSA. Plate was incubated in the dark overnight and then read by ELISA Reader (PerkinElmer, United States). Wells containing the substrate and the enzyme without compounds were used as reaction control. Wells containing biotinylated poly-GluTyr (4:1) and enzyme without ATP were used as basal control. Percent inhibition was calculated by the comparison of compounds treated to control incubations. The concentration of the test compound causing 50%inhibition (IC50) was calculated from the concentration–inhibition response curve (triplicate determinations) and the data were compared with Sorafenib as standard VEGFR-2 inhibitor.

#### 4.2.2. *In vitro* HUVEC anti-proliferative assay

The assay was performed at single dose concentration of 10 µM, where HUVEC, human (Life Technologies # C-003-5C) served as the cells’ source, in Medium 200 (Life Technologies # M-200–500), with large vessel endothelial supplement (LVES) (Life Technologies # A14608-01) and Pen-strep (Hyclone # SV30010). Alamar Blue (Life Technologies # DAL1025) was used as the fluorescent reagent.

HUVEC cells were cultured in Medium 200 with 2% LVES and 1% Pen-strep. To perform the proliferation assay, HUVEC cells were seeded at 5000 cells/50 μl/well in a 96-well black clear bottom tissue culture plate. Cells were incubated at 37 °C and 5% CO2 overnight to allow them to recover and reattach. Next day cells were treated with test compounds for 72 h. After treatment, cell proliferation was measured by Fluorescent quantitation of alamar Blue reagent. The alamar Blue assay incorporates a fluorometric/colorimetric growth indicator based on detection of metabolic activity. Specifically, resazurin, the active ingredient in the alamar Blue reagent, is blue in colour and virtually non-fluorescent. Upon entering cells, resazurin is reduced to resorufin, a compound that is red in colour and highly fluorescent. Continued cell growth maintains a reduced environment, therefore increasing the overall fluorescence and colour of the media surrounding cells. The fluorescence intensity of alamar Blue reagent was shown to be directly proportional to cell number. To perform the alamar Blue assay, 10 µM of alamar Blue reagent was added to each well and the plate was incubated at 37 °C for an additional 2 h. Fluorescence intensity was measured at an excitation of 530 nm and an emission of 590 nm using a BioTek SynergyTM 2 microplate reader.

Cell proliferation assays were performed in triplicate at each concentration. The fluorescent intensity data were analysed using the computer software, Graphpad Prism. In the absence of the compound, the fluorescent intensity (Ft) in each data set was defined as 100%. In the absence of cells, the fluorescent intensity (Fb) in each data set was defined as 0%. The percent cell in the presence of each compound was calculated according to the following equation: % Cell = (F – Fb)/(Ft – Fb), where F = the fluorescent intensity in the presence of the compound, Fb = the fluorescent intensity in the absence of cells, and Ft = the fluorescent intensity in the absence of the compound.

#### 4.2.3. *In vitro* cytotoxic activity

The cytotoxicity assays were performed at NCI, Bethesda, United States (against 56 cell lines). The human tumour cell lines of the cancer screening panel were grown in RPMI 1640 medium containing 5% foetal bovine serum and 2 mM L-glutamine. For a typical screening experiment, cells were inoculated into 96 well microtiter plates in 100 μ at plating densities ranging from 5000 to 40,000 cells/well depending on the doubling time of individual cell lines. After cell inoculation, the microtiter plates were incubated at 37 °C, 5% CO2, 95% air, and 100% relative humidity for 24 h prior to addition of experimental drugs. After 24 h, two plates of each cell line were fixed *in situ* with trichloroacetic acid (TCA), to represent a measurement of the cell population for each cell line at the time of drug addition (*Tz*).

Experimental drugs were solubilised in dimethyl sulfoxide at 400-fold the desired final maximum test concentration and stored frozen prior to use. At the time of drug addition, an aliquot of frozen concentrate was thawed and diluted to twice the desired final maximum test concentration with complete medium containing 50 μg/ml gentamicin. Additional four, 10-fold or ½ log serial dilutions were made to provide a total of five drug concentrations plus control. Aliquots of 100 μl of these different drug dilutions were added to the appropriate microtiter wells already containing100 μl of medium, resulting in the required final drug concentrations. Triplicate wells were prepared for each individual dose. Following drug addition, the plates were incubated for an additional 48 h at 37 oC, 5% CO2, 95% air, and 100% relative humidity. For adherent cells, the assay was terminated by the addition of cold TCA. Cells were fixed *in situ* by the gentle addition of 50 μl of cold 50% (w/v) TCA (final concentration, 10% TCA) and incubated for 60 min at 4 °C. The supernatant was discarded, and the plates were washed five times with tap water and air dried. SRB solution (100 μl) at 0.4% (w/v) in 1% acetic acid was added to each well, and plates were incubated for 10 min at room temperature. After staining, unbound dye was removed by washing five times with 1% acetic acid and the plates were air dried. Bound stain was subsequently solubilised with 10 mM trizma base, and the absorbance was read on an automated plate reader at a wavelength of 515 nm. For suspension cells, the methodology was the same except that the assay was terminated by fixing settled cells at the bottom of the wells by gently adding 50 μl of 80% TCA (final concentration, 16% TCA). Using the seven absorbance measurements [time zero (*T_z_*), control growth (*C*), and test growth in the presence of drug at the five concentration levels (*Ti*)], the percentage growth was calculated at each of the drug concentration levels. Percentage GI was calculated as: [(*Ti – Tz*)/(*C – Tz*)] × 100 for concentrations for which *Ti ≥ Tz* [(*Ti – Tz*)/*Tz*] × 100 for concentrations for which *Ti < Tz*.

Three dose response parameters were calculated for each experimental compound: GI of 50% (GI50) was calculated when [(*Ti – Tz*)/(*C – Tz*)] × 100 = 50.

### 4.3. Methods and computational details

#### 4.3.1. Protein and ligand preparation

The atomic coordinates of the kinase domain of VEGFR-2 co-crystallised with the FDA approved competitive kinase inhibitor, Sorafenib^43^, were retrieved from the protein data bank (PDB ID: 3WZE)^44^. The missing non-terminal segments of the protein were built using MODELLER^45^ using the standard protocol. The structure with the lowest z-DOPE score was selected for further structural investigation and simulation. The z-DOPE score is a normalised atomic distance-dependent statistical potential based on known protein structures. The selected structure was further prepared using the protein preparation wizard in Maestro^46^. In protein preparation, original hydrogen atoms, if any, were deleted and new hydrogen atoms were added. This was followed by adjusting the bond orders for the amino acid residues and the ligand. In adding hydrogen atoms, the protonation states of titrable residues were assigned at pH of 7 using PROPKA51. Finally, a restrained minimisation with convergence of heavy atoms to an RMSD of 0.3 Å was performed utilising the OPLS3^47^ forcefield parameters.

For the ligand preparation step, the chemical structures of **11c** and **18b** were drawn in Maestro. The structures were prepared through the ligprep module of Schrodinger^48^. Ligand preparation includes adding missing hydrogen atoms and assigning proper bond orders and protonation states at pH 7. This was followed by energy minimisation of the compounds in the OPLS3 force field.

#### 4.3.2. Docking and scoring simulations

Three molecules were selected for molecular docking simulations; namely, Sorafenib, compound **11c**, and compound **18b**. The molecular docking simulations were performed using Smina, a version of AutoDock Vina optimised to support high-throughput minimisation and scoring, and also provides enhanced control over docking parameters^49^^,^^50^. The docking search space was centred on the centre of mass of the original ligand of the 3WZE structure, Sorafenib, and extended to 20 Å × 20 Å × 20 Å box dimension. The best 5 poses for each ligand were rescored through three different scoring functions, including NNscore 2^51^, RFscore^52^, and DLSCORE^53^. For each ligand, the pose that showed the smallest heavy atoms RMSD compared to the co-crystallised ligand, Sorafenib was selected to carry out the MD simulation and binding energy calculations.

#### 4.3.3. Classical molecular dynamics simulations

All MD simulations were carried out in AMBER14 software package for the ligand pose that showed the smallest RMSD value to the co-crystallised ligand (see above). Each complex was immersed in a cubic box of TIP3P water model and neutralised by counter ions. Final salt concentration was brought to 150 mM NaCl concentration to mimic physiological conditions. Each solvated system was minimised through four steps: first, a harmonic constraint potential of 100 kcal/mol/Å^2^ was applied to all non-hydrogen atoms of the protein and the ligand. In the following minimisation stages, the restrains were reduced to 50, 5, and 0 kcal/mol/Å^2^. In each step, minimisation was performed by the steepest descent method for the first 1000 steps and the conjugated gradient method for the subsequent 4000 steps. Each system was then gradually heated in the NVT ensemble from 0 to 300 K in 100 ps using a Langevin thermostat with a coupling coefficient of 1.0/ps with a force constant 5.0 kcal/mol/Å^2^ on the atoms of the protein and the ligand, using a 1 fs integration time step. And then additional two rounds of MD (50 ps each at 300 K) were performed with decreasing protein/ligand heavy atoms restraint weights from 1, 0.5 to 0.1 kcal/mol/Å^2^. Finally, each system was again equilibrated for 1000 ps by releasing all restrains. Actual MD production run was performed for 6 × 5 ns in the NPT ensemble for each system giving a total production simulation of 30 ns. The first 5 ns was discarded from our analysis and considered as a final equilibration phase. All equilibration and production simulations were performed at 300 K with Berendsen temperature coupling^54^ and 1 atm with isotropic molecule-based scaling and a 2fs integration time step. Long-range Coulombic interactions were handled using the Particle Mesh Ewald method^55^. The cutoff distance for the electrostatic and vdW energy term was set at 9.0 Å. To avoid edge effects in all calculations, Periodic boundary conditions were applied. To allow for an integration time step of 2 fs, the SHAKE algorithm^56^ was applied for all bonds involving hydrogen atoms. Trajectory frames were recorded every 4 ps throughout all production runs. All analysis script was written in python and bash and performed mainly through the CPPTRAJ utility in AMBER^57^.

#### 4.3.4. The AMBER/MM-GBSA binding energy calculation

The final 25 ns MD simulation trajectory was used to estimate the free binding energy using the AMBER/MM-GBSA method^58^. A total number of 3101 frames with an 8 ps interval were selected to perform the binding energy analysis. All waters and counter ions were stripped prior to carrying out the binding energy calculations. The MM-GBSA protocol was carried out similarly for all protein-ligand complexes. In summary, the MMG-GBSA protocol approximates the free binding energy (Δ*G*_bind_) as follow:

$$\Delta G_{\text{bind}}=G_{\text{complex}}–\left(G_{\text{protein}}+G_{\text{ligand}}\right)$$

$$\Delta G_{\text{bind}}=\Delta E_{\text{MM}}+\Delta G_{\text{solv}}–T\Delta S$$

$$=\Delta E_{\text{vdW}}+\Delta E_{\text{ELE}}+\Delta G_{\text{pol}}+\Delta G_{\text{nonpol}}–T\Delta S$$

In the previous formulas, *G*_complex_ is the protein-ligand complex free energy, *G*_protein_ is the protein free energy, and *G*_ligand_ is the ligand free energy. Δ*G*_bind_ is approximated by summing the changes in the MM (Δ*E*_MM_) energy, the solvation energy (Δ*G*_solv_), and the entropic contributions (*T*Δ*S*). Δ*E*_MM_ is further divided by lipophilic vdW (Δ*E*_vdW_) and electrostatic (Δ*E*_ELE_) components. The solvation energy (Δ*G*_solv_) is further divided into polar (Δ*G*_pol_) and non-polar (Δ*G*_nonpol_) contributions. The vdW (Δ*E*_vdW_) and electrostatic (Δ*E*_ELE_) components were extracted from the MD simulation trajectory using the same forcefield parameters. The polar component (Δ*G*_pol_) of the solvation energy was calculated using the GBSA module of AMBER. The non-polar component was estimated as Δ*G*_nonpol_ = *γ*SASA + *β*, SASA is the solvent-accessible surface area, *γ* and *β* are two constants that were set to 0.0072 kcal/mol/Å and 0.0 kcal/mol, respectively. For practical reasons, the last term (***TΔS***) was ignored.

#### 4.3.5. Relaxed PES scan

The three-dimensional structure of compounds **11c and 18b** were superposed on top of the bioactive, co-crystallised conformation of Sorafenib. Upon docking, we observed that the majority of the generated docked poses of 18b indeed suggests that this conformation as the optimal binding pose of **18b**. For compound **11c**, however, none of the generated poses exhibited this conformation. At this stage, it was not clear whether this was a shortcoming from the sampling algorithm of the docking or simply because this pose was very energetically unfavourable. This may explain the low activity of compound **11c** even though it bears the required H-bond acceptor site at the terminal hetero-aromatic ring. To investigate this effect in more details, we ran a relaxed 2D PES scan for the terminal ring torsion depicted in Figure 6 (C-C-X-C, where X is an O atom in SORA and **18b,** and N **in 11c**). Performing a PES scan at the MM level of theory is impractical given the inherent approximations at this level of theory. Alternatively, we decided to perform the PES scan at the QM level of theory using a Density Functional Theory Based model. The starting conformation for the PES scans for all ligands was the Sorafenib bound like ligand conformation. PES scan was performed at the B3LYP/6–31 + G* level of theory, as employed in Gaussian 16 program package^59^. Selected torsional angle was rotated by 360° over 36 scan steps; i.e. 10° increment for each step. For each ligand, identified minima on the PES were further optimised at the M062X/6–311++G** level of theory. Frequency calculations at the same model (M062X/6–311++G**) showed that all frequencies are positive, which indicates that the identified minima are true minima on the PES surface.

## 5. Conclusion

We reported herein a set of pyridazine analogues as potent inhibitors for the VEGFR-2 enzyme. Compounds **8c, 8f, 15, 18b, and 18c** emerged as the most potent VEGFR-2 inhibitors in this study with IC50 values ranging from low micromolar to low nanomolar ranges. Among these inhibitors, we reported compound **18b** as a potent nano-molar inhibitor (60.7 nm IC_50_) of VEGFR-2, comparable to the well-established FDA approved competitive kinase inhibitor, Sorafenib. Cellular activity studies showed that compound **18b** exhibited a potent anti-proliferative activity against the HUVEC cell line *in vitro*. Most likely, the compounds target the DFG-out conformation of VEGFR-2 and occupying the nearby allosteric pocket.

Detailed analysis of the binding modes and low energy conformational preferences of two of the novel compounds (compounds **11c** and **18b**) together with the co-crystallised compound, sorafenib (SORA) revealed that certain features are required to observe an acceptable activity profile. First the presence of the hinge H-bond with CYS919 is a prerequisite for the VEGFR-2 inhibition activity. Second; an accessible low energy conformation that can orient the necessary binding features to the binding site residues is a must to achieve good inhibition. As we discussed in compound **11c**, although the molecule indeed contains an H-bond acceptor centre at the terminal hetero-aromatic ring, the conformation necessary to orient this H-bond in the proper place is strained, hence very energetically unfavourable (∼3.0 kcal/mol from the favourable planar global minimum conformation). Furthermore, the calculated free binding energies and ligand stabilities in the binding site reflected the observed activity trend.

Furthermore, seven selected compounds **3, 5a–c, 8a–f, 11a–f, 15,** and **18a–c** were evaluated for their *in vitro* anticancer activity according to US-NCI protocol. The results revealed that compound **11f** featuring a 3-chloropyridazine head group as well as a lipophilic 3-trifloromethyl 4-chloro on the pendant phenyl which elicited the highest activity (mean % GI = 45). It exhibited broad spectrum and potent anti-proliferative activity against several NCI cell panel. Interestingly, compound **11f** exhibited poor VEGFR2 inhibition activity. Further investigations are required to determine the mechanism of anticancer activity of **11f**.

## Supplementary Material

Supplemental Material
