# Supplementary material for: Towards discovery of novel scaffold with potent antiangiogenic activity; design, synthesis of pyridazine based compounds, impact of hinge interaction, and accessibility of their bioactive conformation on VEGFR-2 activities
Source: J Enzyme Inhib Med Chem. 2019 Sep 6;34(1):1573–89. doi: 10.1080/14756366.2019.1651723 (PMC6746272; doi:10.1080/14756366.2019.1651723)
Supplement: Supplemental Material [file IENZ_A_1651723_SM7639.pdf]

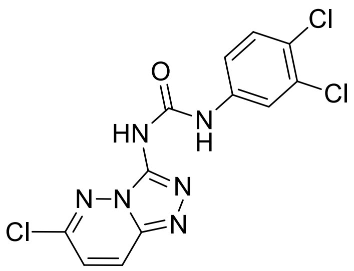
Cl
O
Cl
HN
N
H
N
N
N
N
Cl

Cpd 3

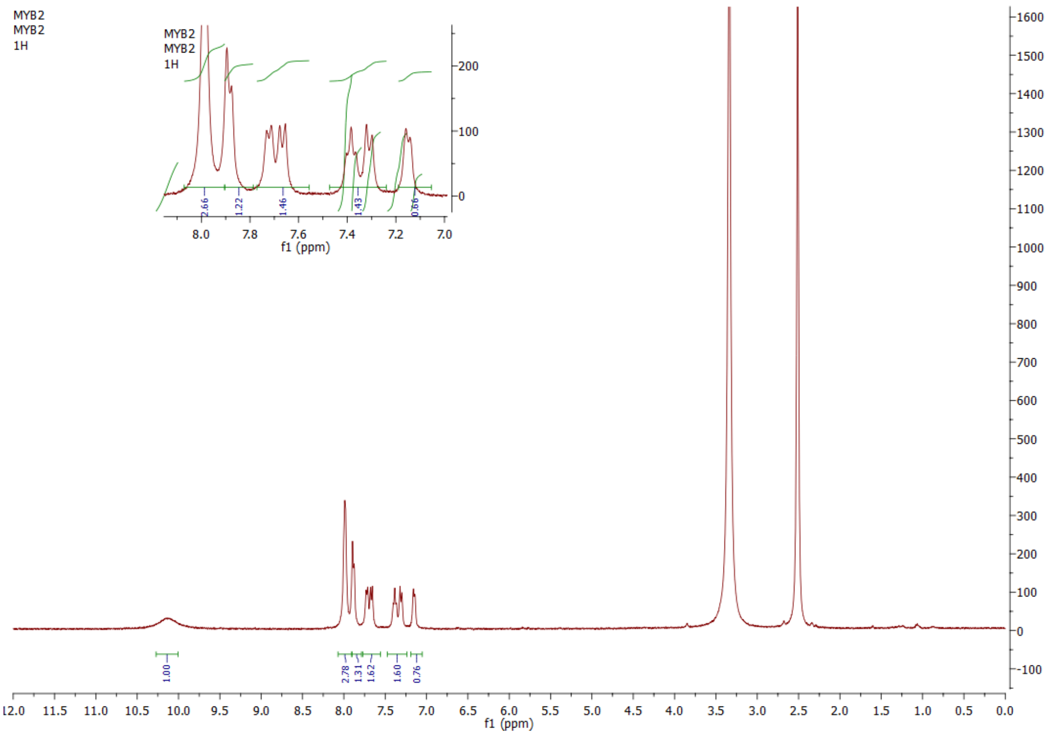
MYB2
MYB2
1H
MYB2
MYB2
1H
2.66
1.22
1.46
1.43
0.68
8.0
7.8
7.6
7.4
7.2
7.0
f1 (ppm)
1.00
2.78
1.31
1.62
1.60
0.76
12.0
11.5
11.0
10.5
10.0
9.5
9.0
8.5
8.0
7.5
7.0
6.5
6.0
5.5
5.0
4.5
4.0
3.5
3.0
2.5
2.0
1.5
1.0
0.5
0.0
f1 (ppm)

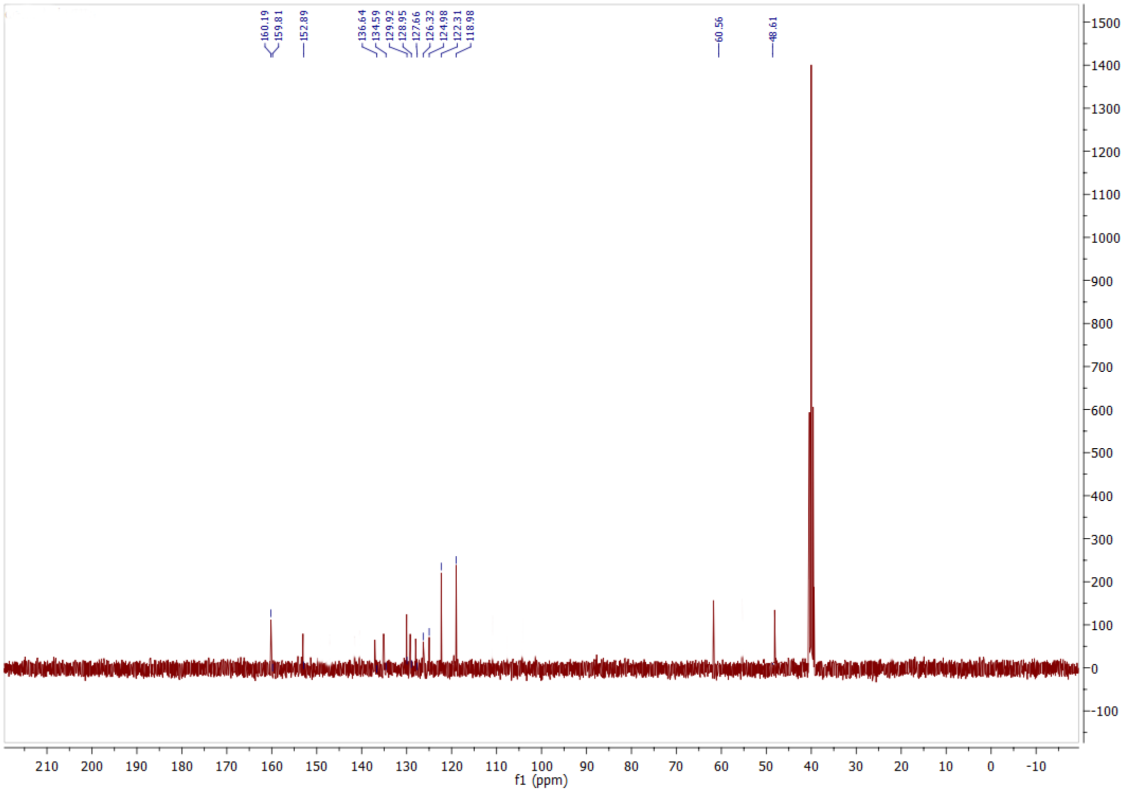
160.19
159.81
152.89
136.64
134.59
129.92
128.95
127.66
126.32
124.98
122.31
118.98
60.56
48.61
f1 (ppm)

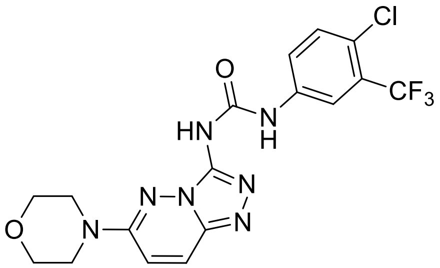

O
HN
N
H
Cl
CF3
N
N
N
N
N
O
N

5c

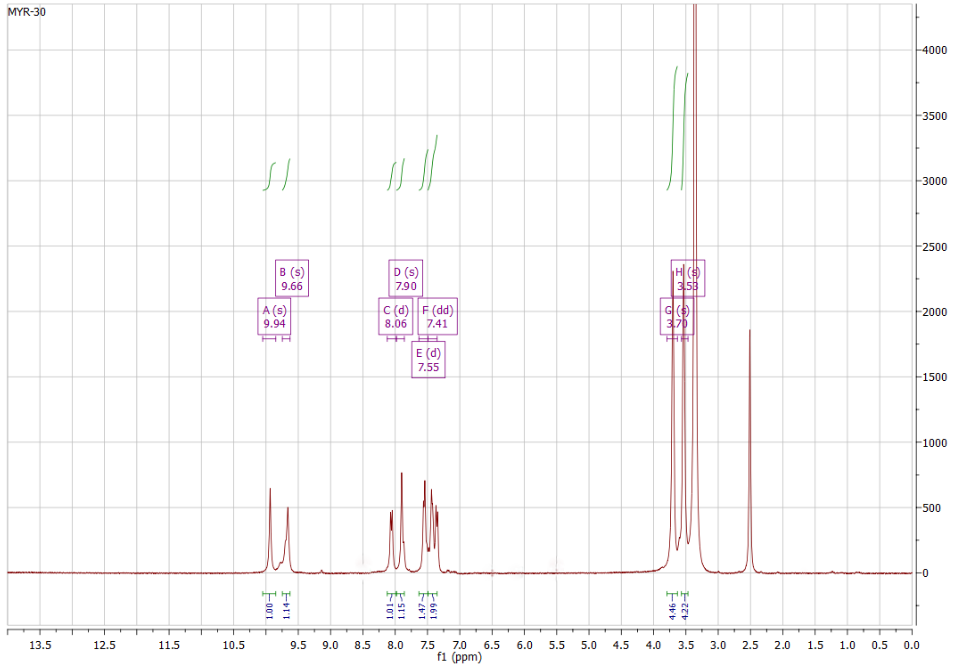

MYR-30
A (s)
9.94
B (s)
9.66
C (d)
8.06
D (s)
7.90
E (d)
7.55
F (dd)
7.41
G (s)
3.70
H (s)
3.53
1.00
1.14
1.01
1.15
1.47
1.99
4.46
4.22
f1 (ppm)

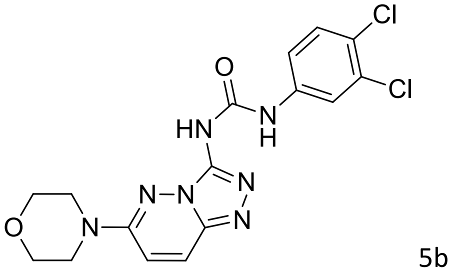

5b

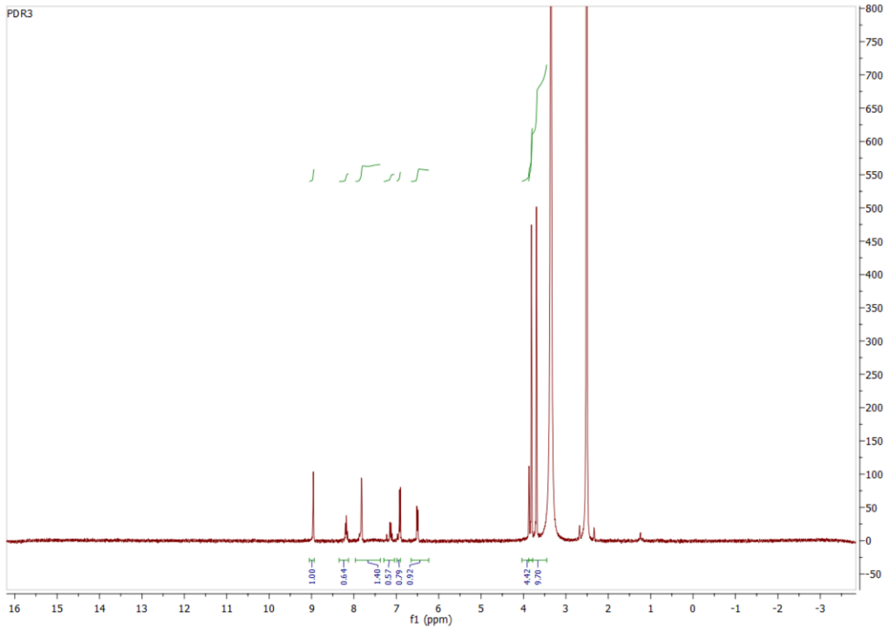

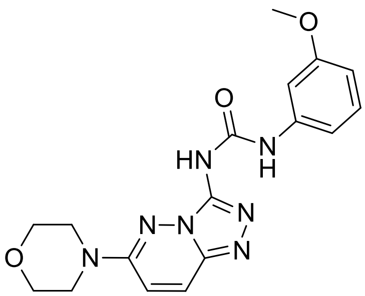

5a

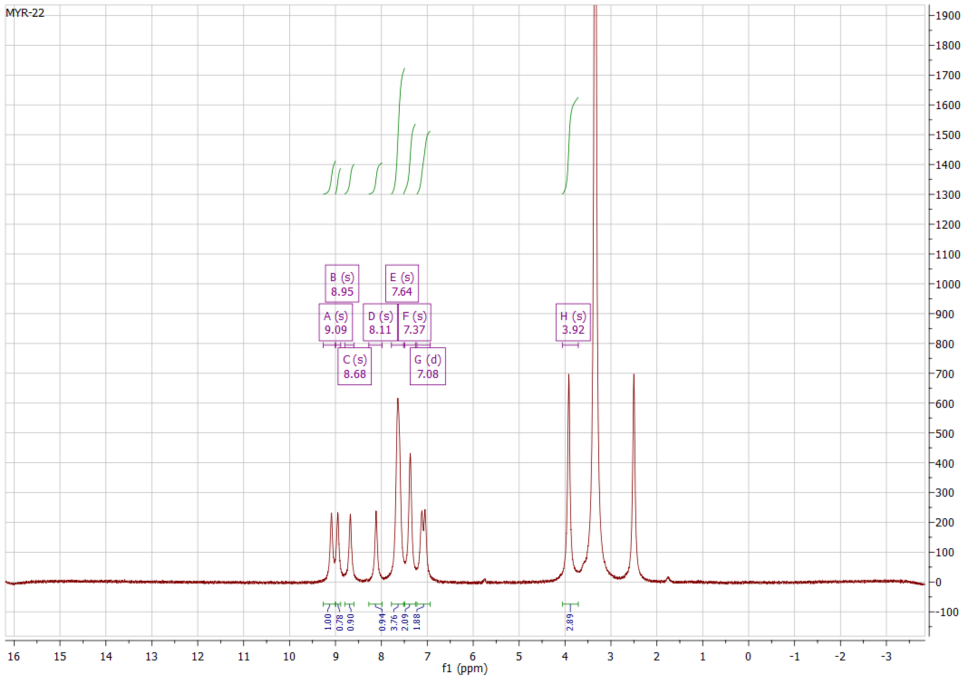
MYR-22
A (s) 9.09
B (s) 8.95
C (s) 8.68
D (s) 8.11
E (s) 7.64
F (s) 7.37
G (d) 7.08
H (s) 3.92
1.00
0.78
0.90
0.94
3.76
2.09
1.88
2.89
f1 (ppm)

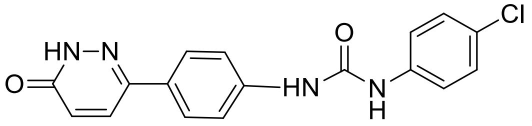

8a

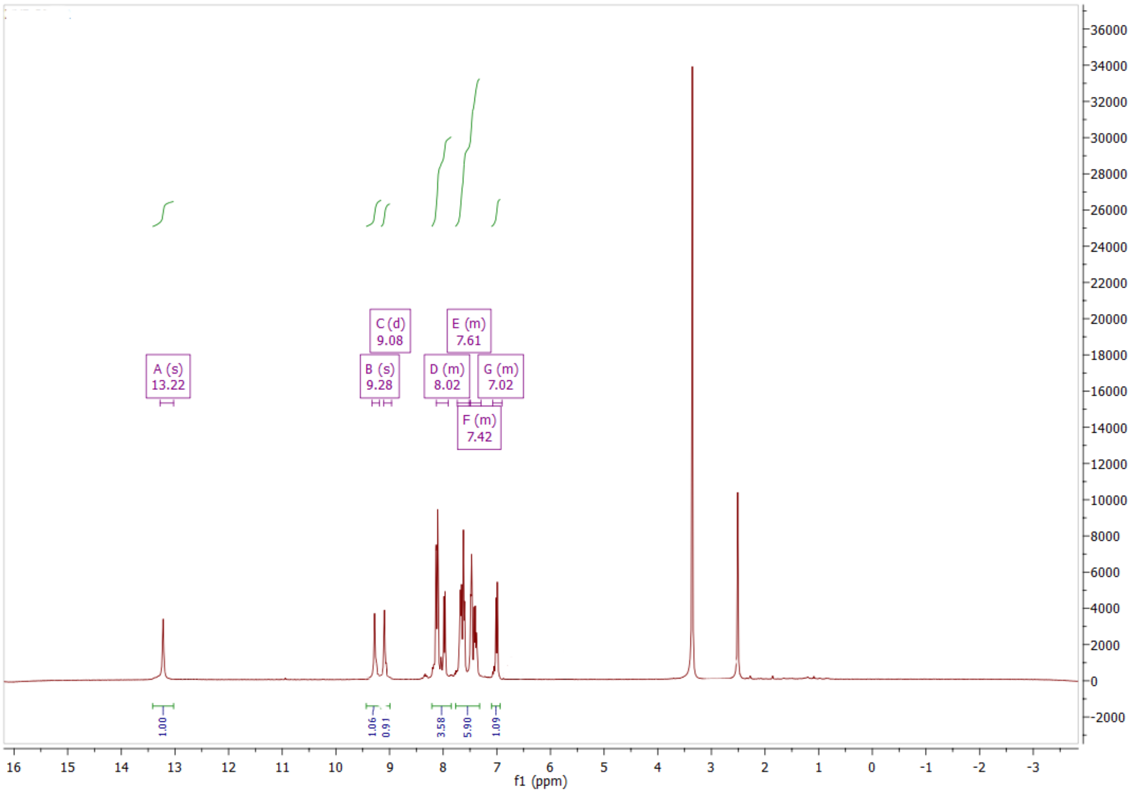

A (s)
13.22
B (s)
9.28
C (d)
9.08
D (m)
8.02
E (m)
7.61
F (m)
7.42
G (m)
7.02
1.00
1.06
0.91
3.58
5.90
1.09
f1 (ppm)

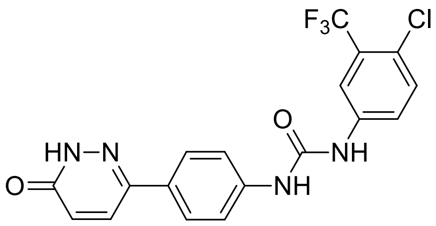

8c

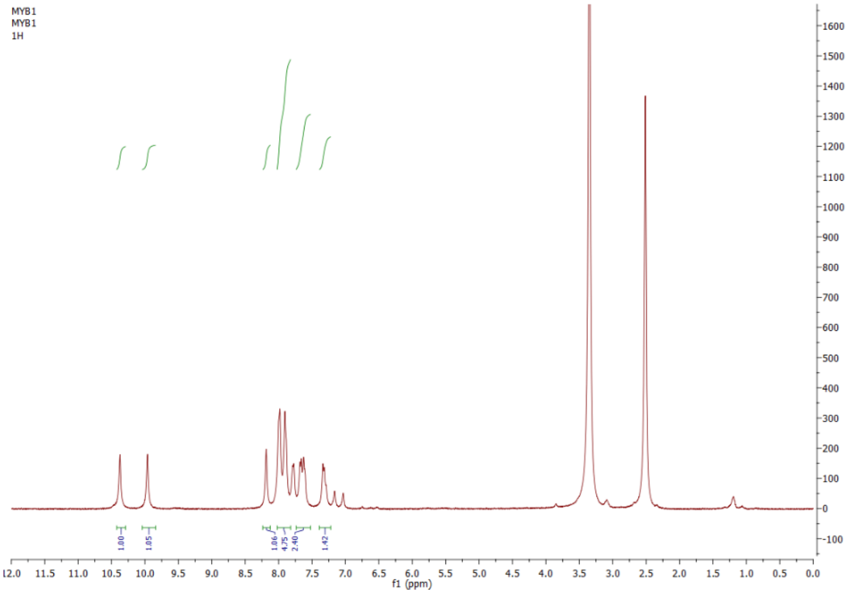

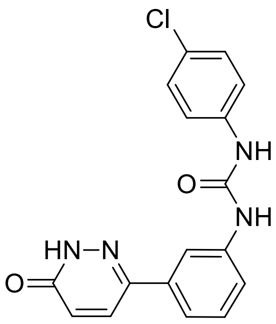

8d

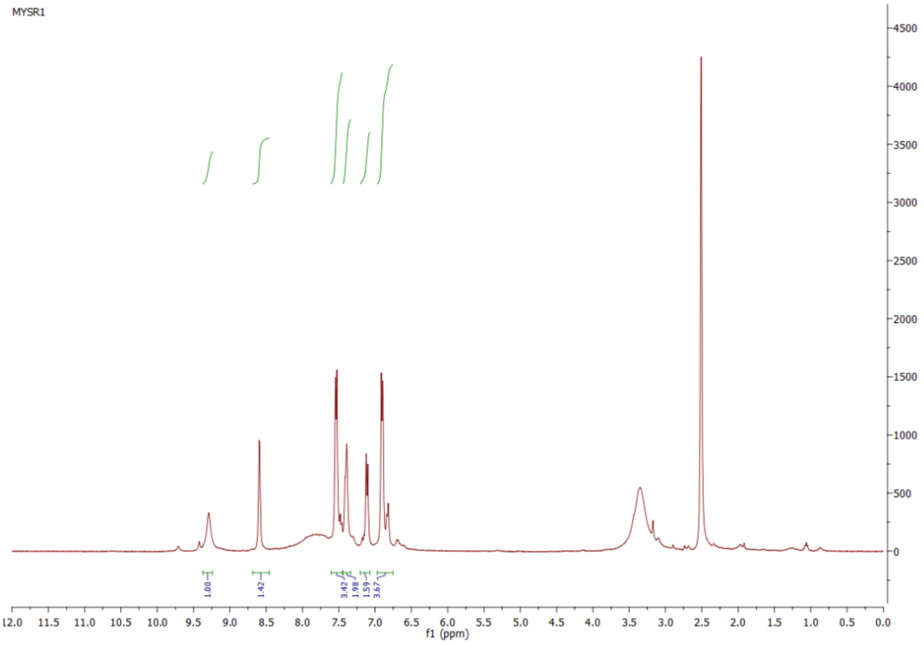

MYSR1
4500
4000
3500
3000
2500
2000
1500
1000
500
0
1.00
1.42
3.42
1.98
1.59
3.67
12.0 11.5 11.0 10.5 10.0 9.5 9.0 8.5 8.0 7.5 7.0 6.5 6.0 5.5 5.0 4.5 4.0 3.5 3.0 2.5 2.0 1.5 1.0 0.5 0.0
f1 (ppm)

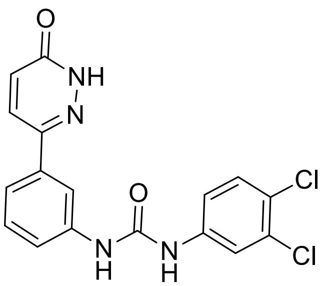

8e

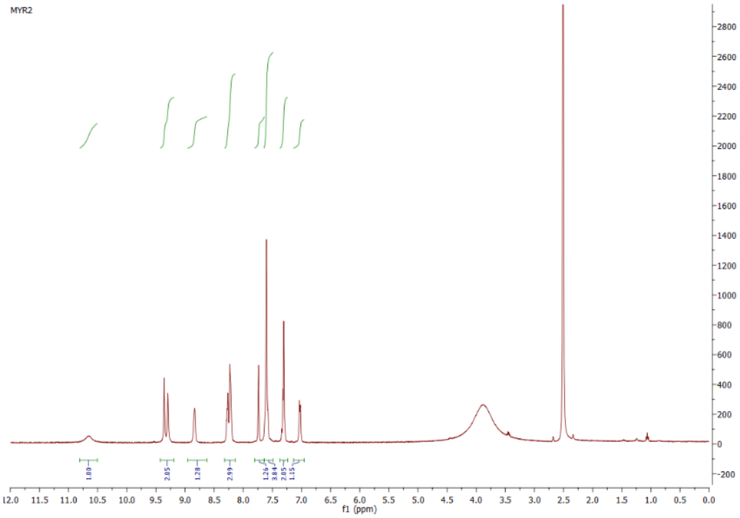

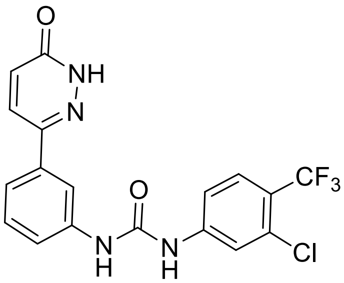

8f

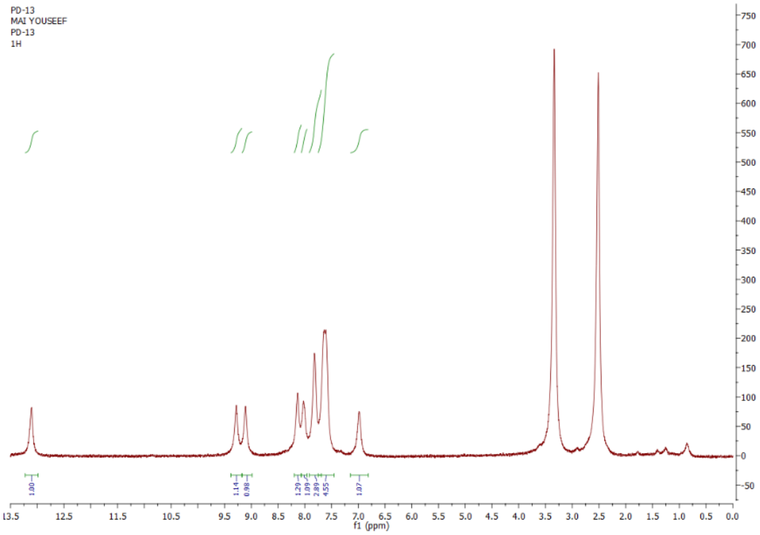

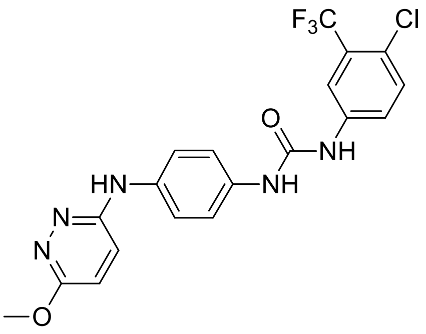
F3C
Cl
O
NH
HN
NH
N
N
O

15

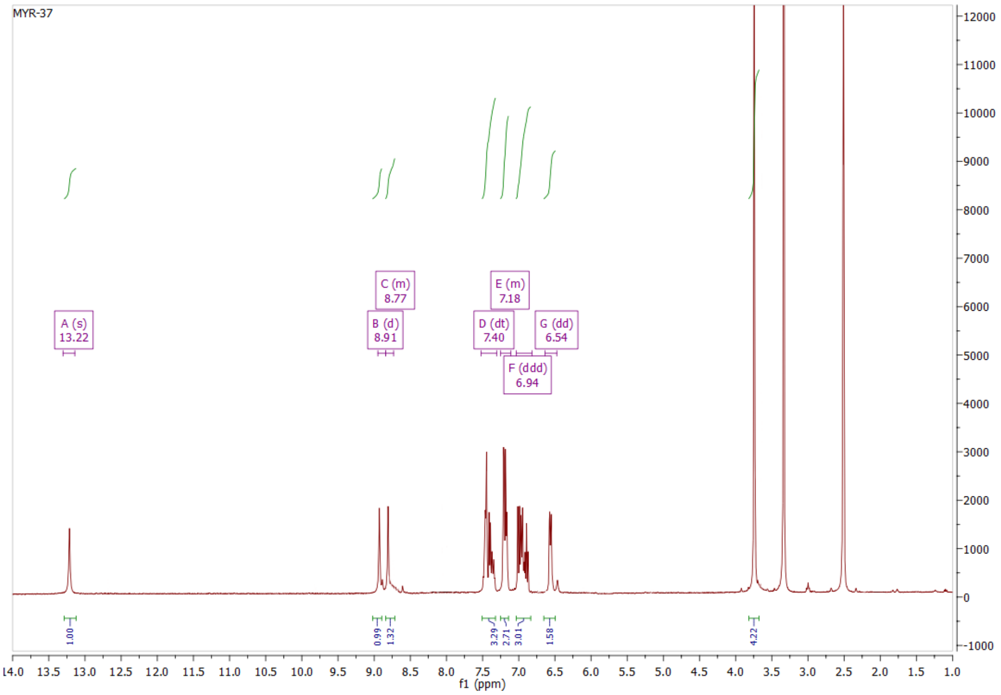
MYR-37
A (s)
13.22
B (d)
8.91
C (m)
8.77
D (dt)
7.40
E (m)
7.18
F (ddd)
6.94
G (dd)
6.54
1.00
0.99
1.32
3.29
2.71
3.01
1.58
4.22
f1 (ppm)

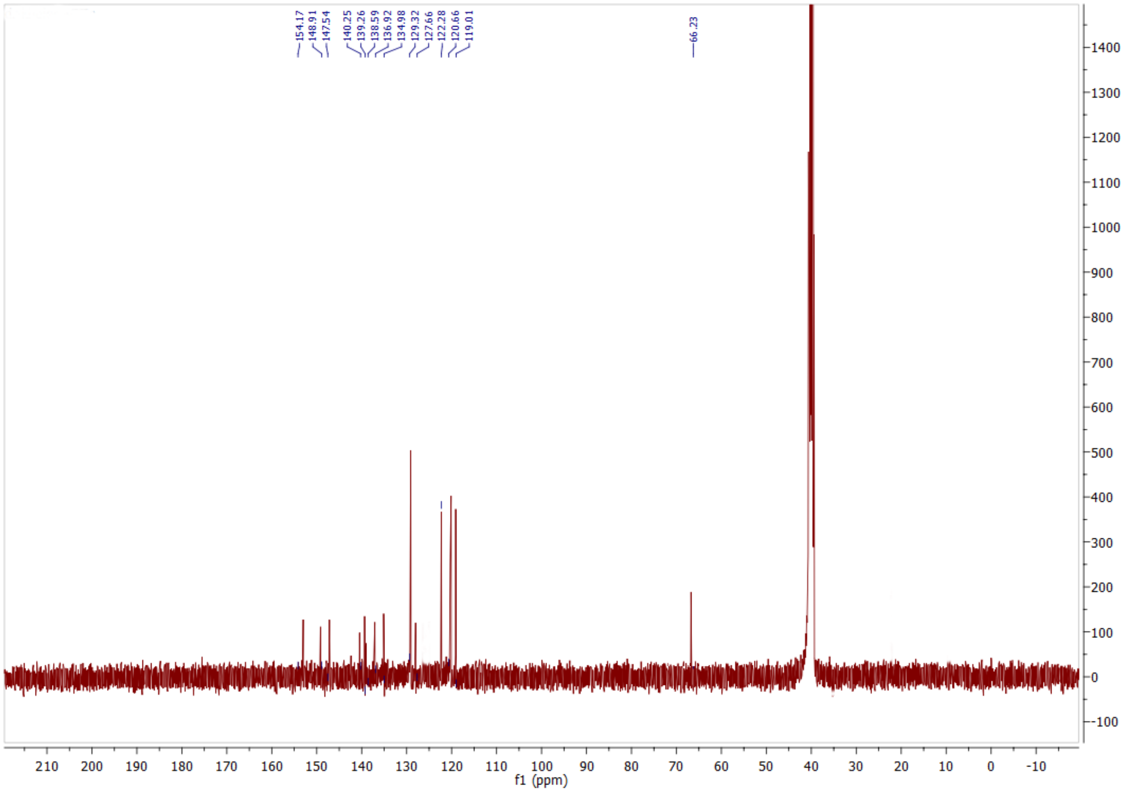

154.17
148.91
147.54
140.25
139.26
138.59
136.92
134.98
129.32
127.66
122.28
120.66
119.01
66.23
f1 (ppm)

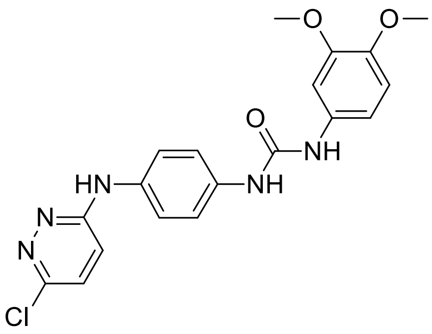

O
O
O
NH
HN
NH
N
N
Cl

11a

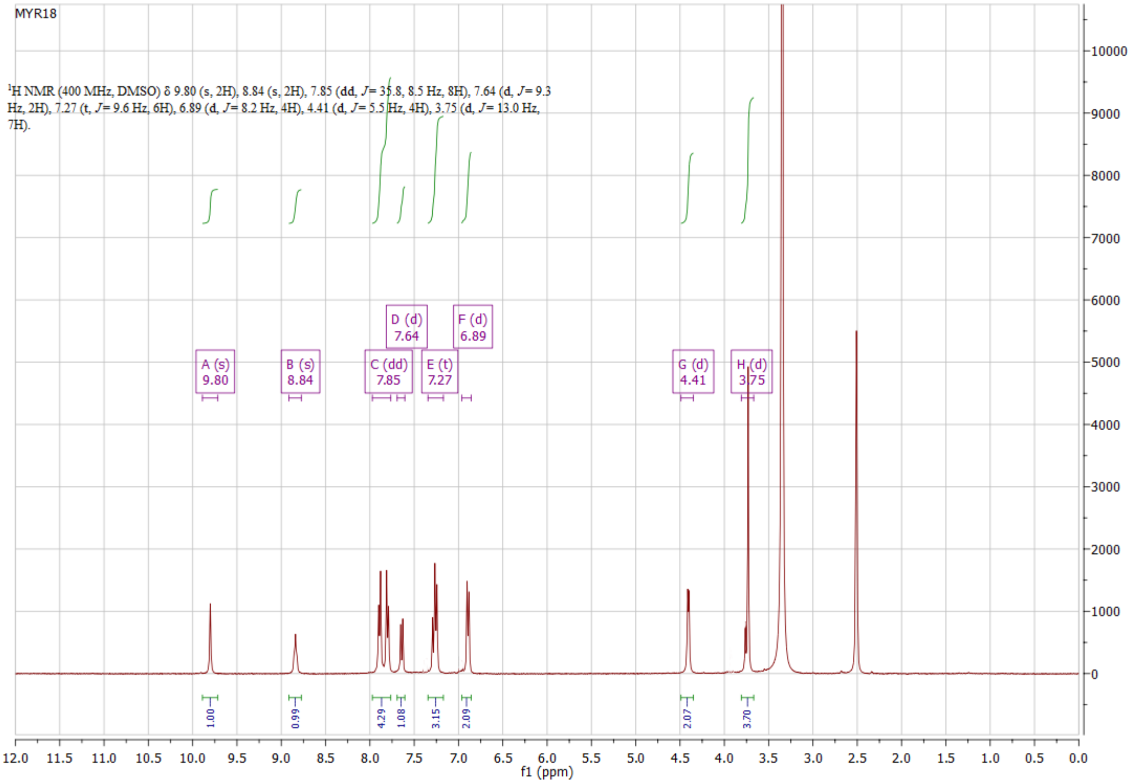

MYR18
1H NMR (400 MHz, DMSO) δ 9.80 (s, 2H), 8.84 (s, 2H), 7.85 (dd, J= 35.8, 8.5 Hz, 8H), 7.64 (d, J= 9.3 Hz, 2H), 7.27 (t, J= 9.6 Hz, 6H), 6.89 (d, J= 8.2 Hz, 4H), 4.41 (d, J= 5.5 Hz, 4H), 3.75 (d, J= 13.0 Hz, 7H).
A (s) 9.80
B (s) 8.84
C (dd) 7.85
D (d) 7.64
E (t) 7.27
F (d) 6.89
G (d) 4.41
H (d) 3.75
1.00
0.99
4.29
1.08
3.15
2.09
2.07
3.70
10000
9000
8000
7000
6000
5000
4000
3000
2000
1000
0
12.0
11.5
11.0
10.5
10.0
9.5
9.0
8.5
8.0
7.5
7.0
6.5
6.0
5.5
5.0
4.5
4.0
3.5
3.0
2.5
2.0
1.5
1.0
0.5
0.0
f1 (ppm)

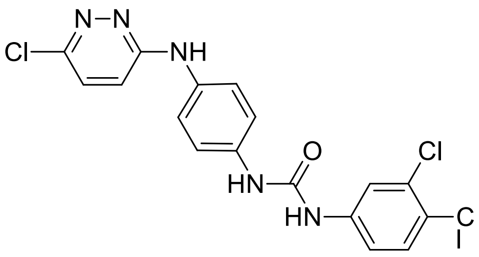
Cl
N
N
NH
HN
O
HN
Cl
C
I

11b

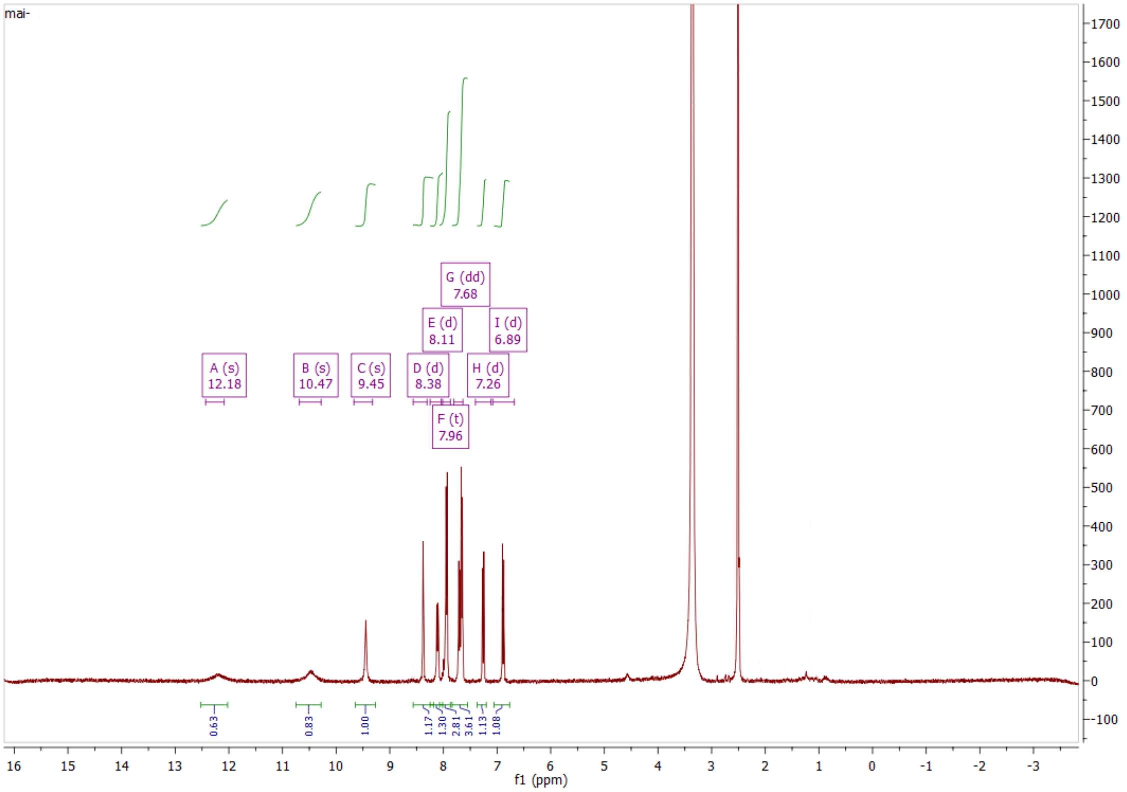
mai-
A (s)
12.18
B (s)
10.47
C (s)
9.45
D (d)
8.38
E (d)
8.11
F (t)
7.96
G (dd)
7.68
H (d)
7.26
I (d)
6.89
0.63
0.83
1.00
1.17
1.30
2.81
3.61
1.13
1.08
f1 (ppm)

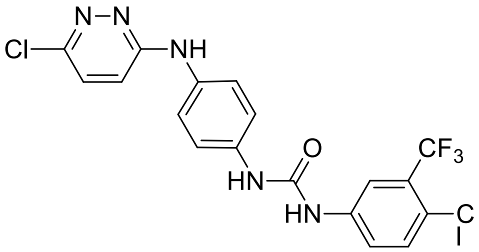
N
N
Cl
NH
O
CF3
HN
HN
C
l

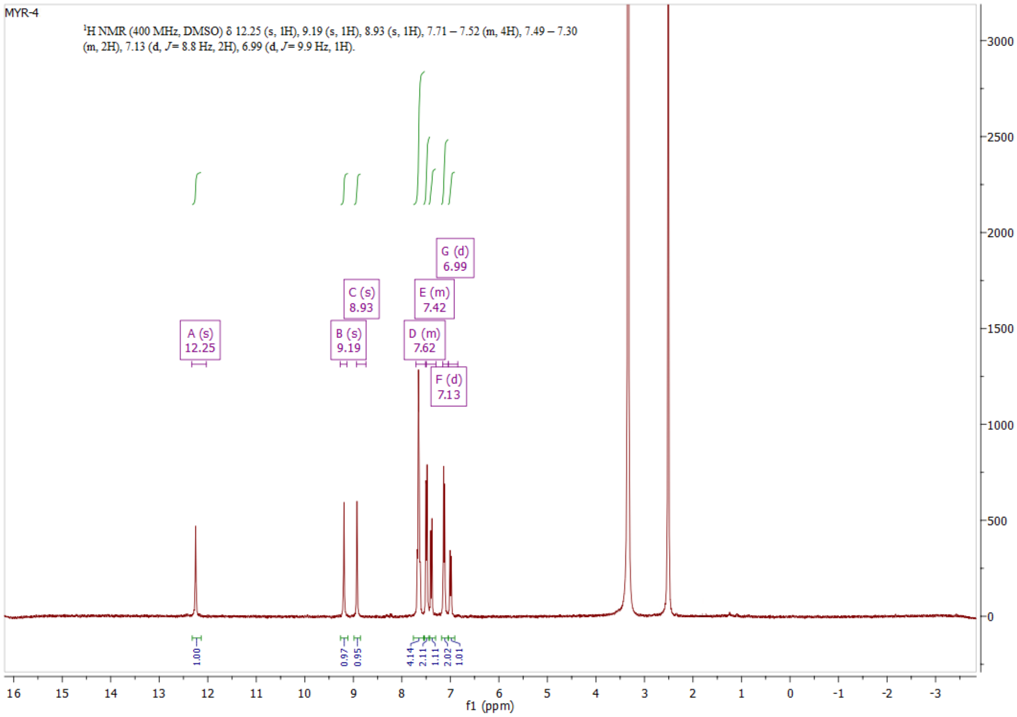
MYR-4
1H NMR (400 MHz, DMSO) δ 12.25 (s, 1H), 9.19 (s, 1H), 8.93 (s, 1H), 7.71 – 7.52 (m, 4H), 7.49 – 7.30 (m, 2H), 7.13 (d, J= 8.8 Hz, 2H), 6.99 (d, J= 9.9 Hz, 1H).
A (s) 12.25
B (s) 9.19
C (s) 8.93
D (m) 7.62
E (m) 7.42
F (d) 7.13
G (d) 6.99
1.00
0.97
0.95
4.14
2.11
1.11
2.02
1.01
f1 (ppm)

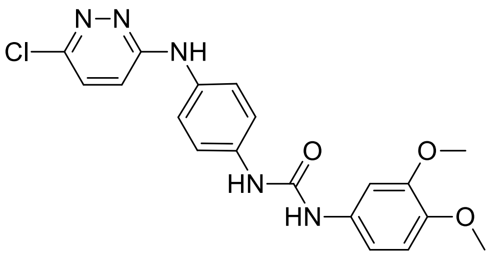

11d

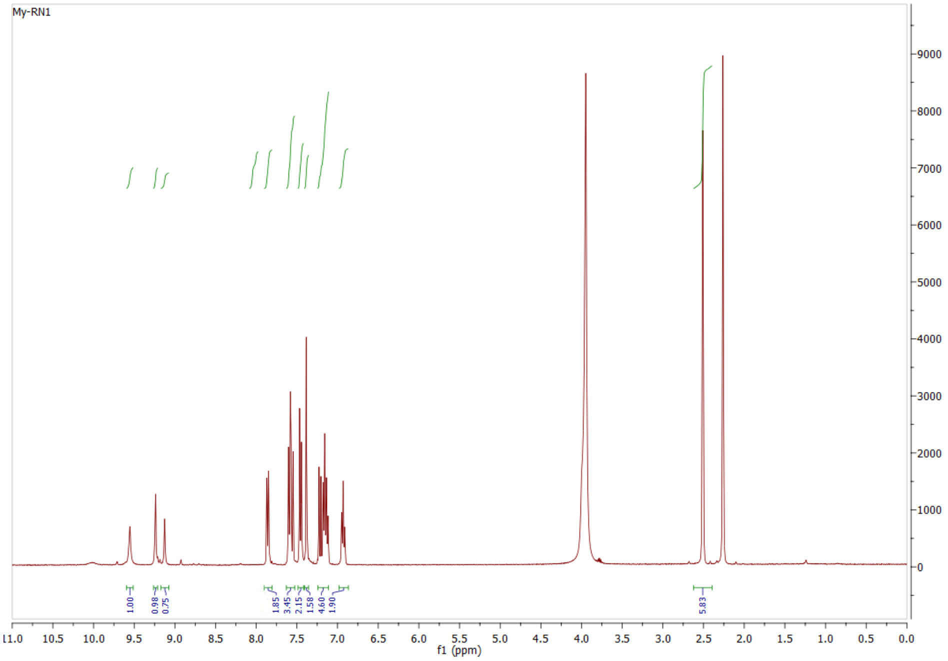
My-RN1
9000
8000
7000
6000
5000
4000
3000
2000
1000
0
1.00
0.98
0.75
1.85
3.45
2.15
1.58
4.60
1.90
5.83
11.0 10.5 10.0 9.5 9.0 8.5 8.0 7.5 7.0 6.5 6.0 5.5 5.0 4.5 4.0 3.5 3.0 2.5 2.0 1.5 1.0 0.5 0.0
f1 (ppm)

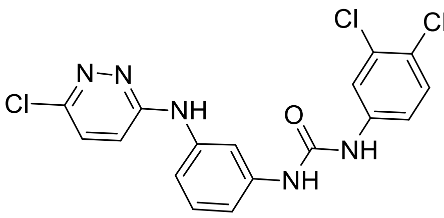
Cl
Cl
N–N
Cl
NH
O
NH
NH

11e

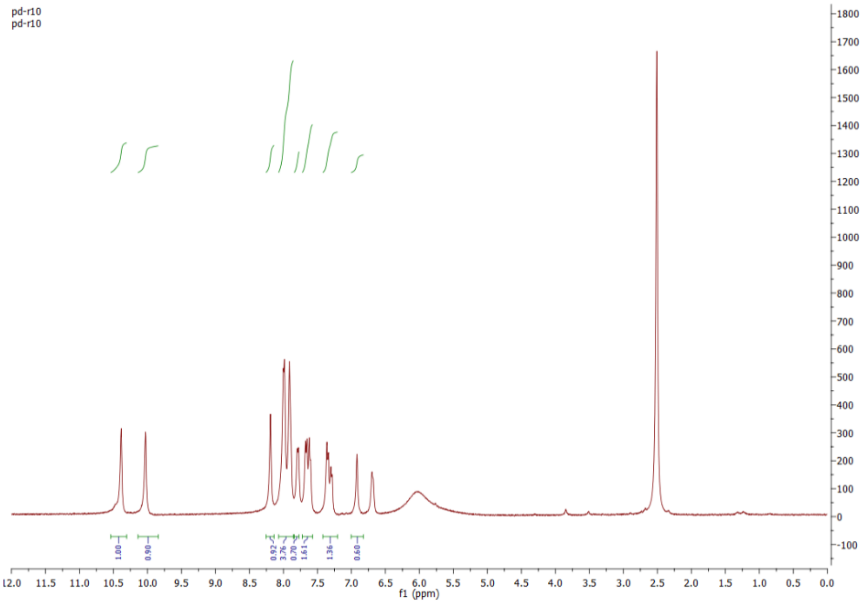
pd-r10
pd-r10
1800
1700
1600
1500
1400
1300
1200
1100
1000
900
800
700
600
500
400
300
200
100
0
-100
12.0 11.5 11.0 10.5 10.0 9.5 9.0 8.5 8.0 7.5 7.0 6.5 6.0 5.5 5.0 4.5 4.0 3.5 3.0 2.5 2.0 1.5 1.0 0.5 0.0
f1 (ppm)

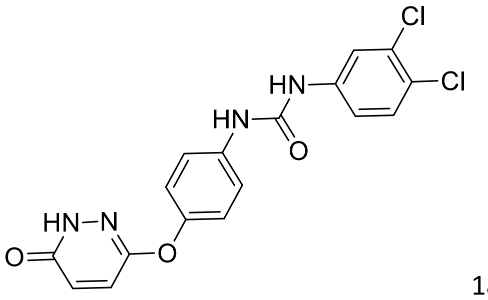

18c

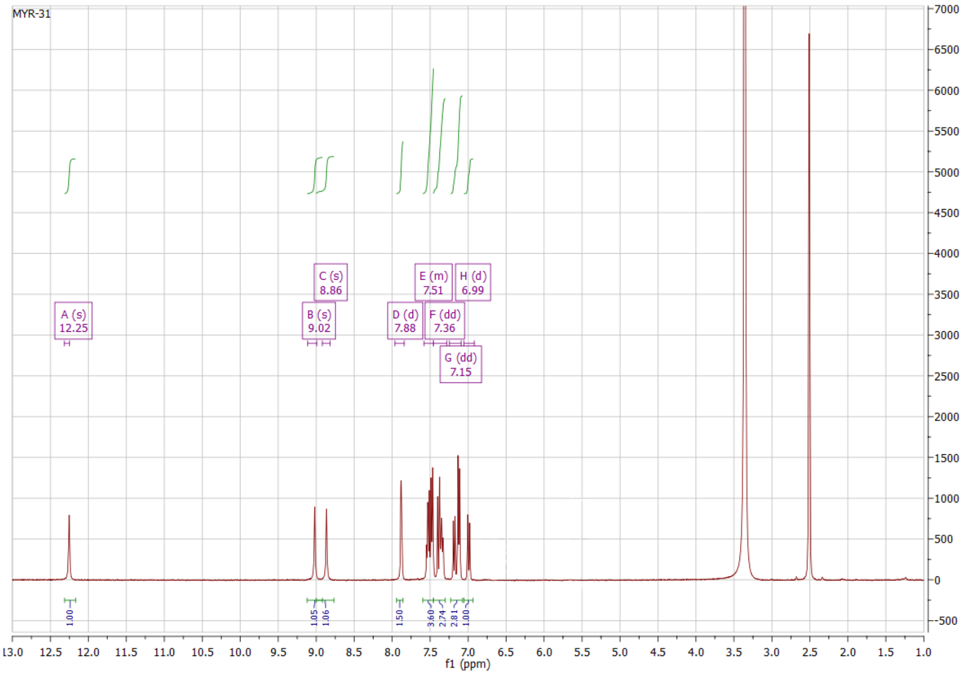
MYR-31
A (s)
12.25
B (s)
9.02
C (s)
8.86
D (d)
7.88
E (m)
7.51
F (dd)
7.36
G (dd)
7.15
H (d)
6.99
f1 (ppm)

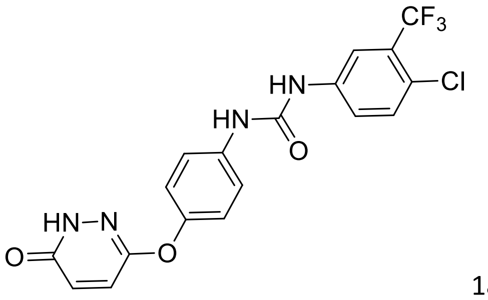
CF3
HN
Cl
HN
O
HN–N
O
O

18b

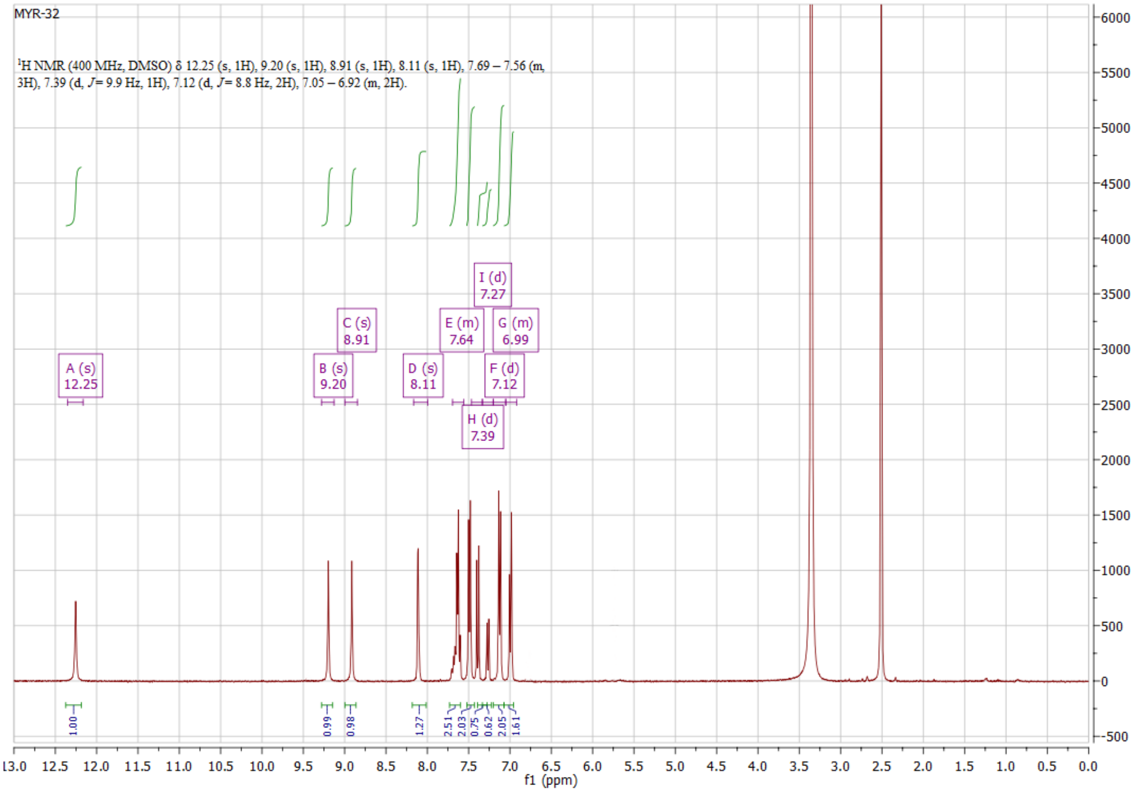
MYR-32
1H NMR (400 MHz, DMSO) δ 12.25 (s, 1H), 9.20 (s, 1H), 8.91 (s, 1H), 8.11 (s, 1H), 7.69 – 7.56 (m, 3H), 7.39 (d, J= 9.9 Hz, 1H), 7.12 (d, J= 8.8 Hz, 2H), 7.05 – 6.92 (m, 2H).
A (s) 12.25
B (s) 9.20
C (s) 8.91
D (s) 8.11
E (m) 7.64
I (d) 7.27
G (m) 6.99
F (d) 7.12
H (d) 7.39
1.00
0.99
0.98
1.27
2.51
2.03
0.75
0.62
2.05
1.61
f1 (ppm)

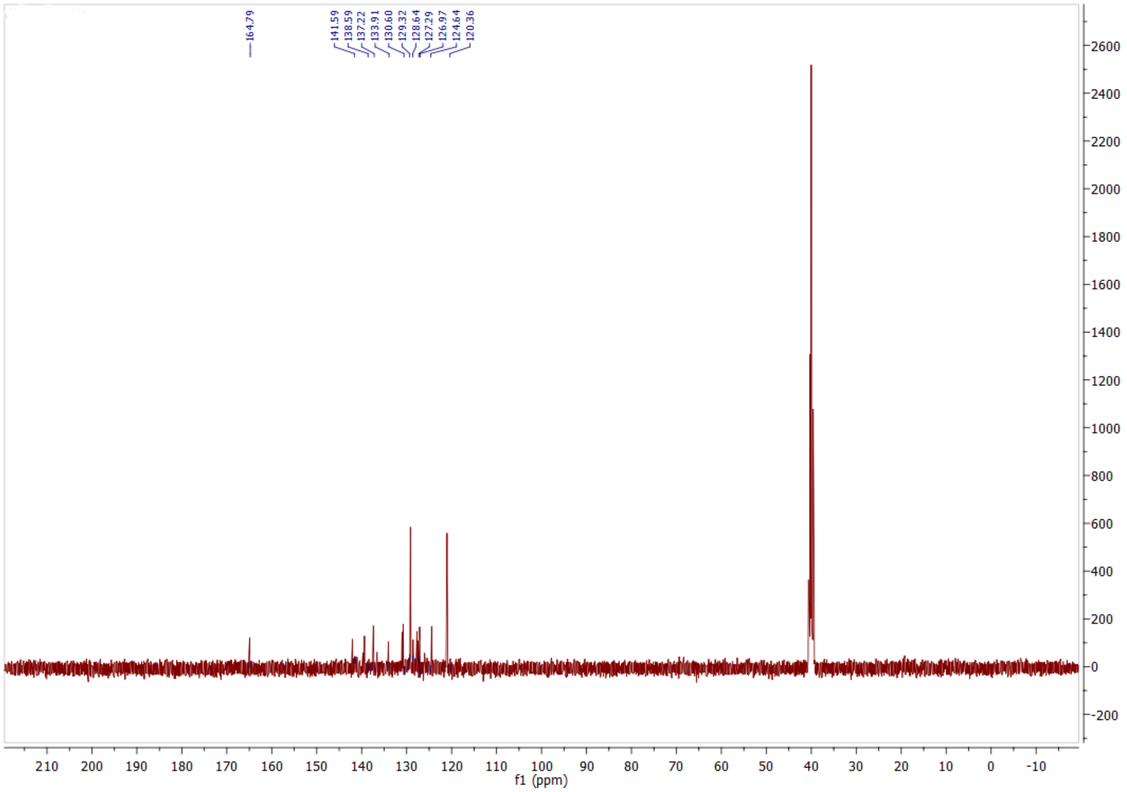

164.79
141.59
138.59
137.22
133.91
130.60
129.32
128.64
127.29
126.97
124.64
120.36
2600
2400
2200
2000
1800
1600
1400
1200
1000
800
600
400
200
0
-200
210 200 190 180 170 160 150 140 130 120 110 100 90 80 70 60 50 40 30 20 10 0 -10
f1 (ppm)

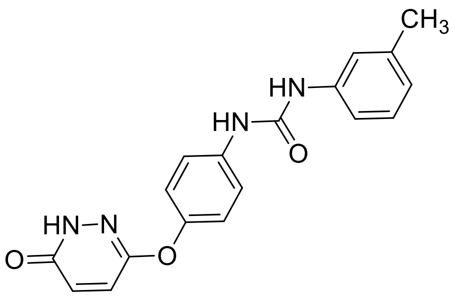

18a

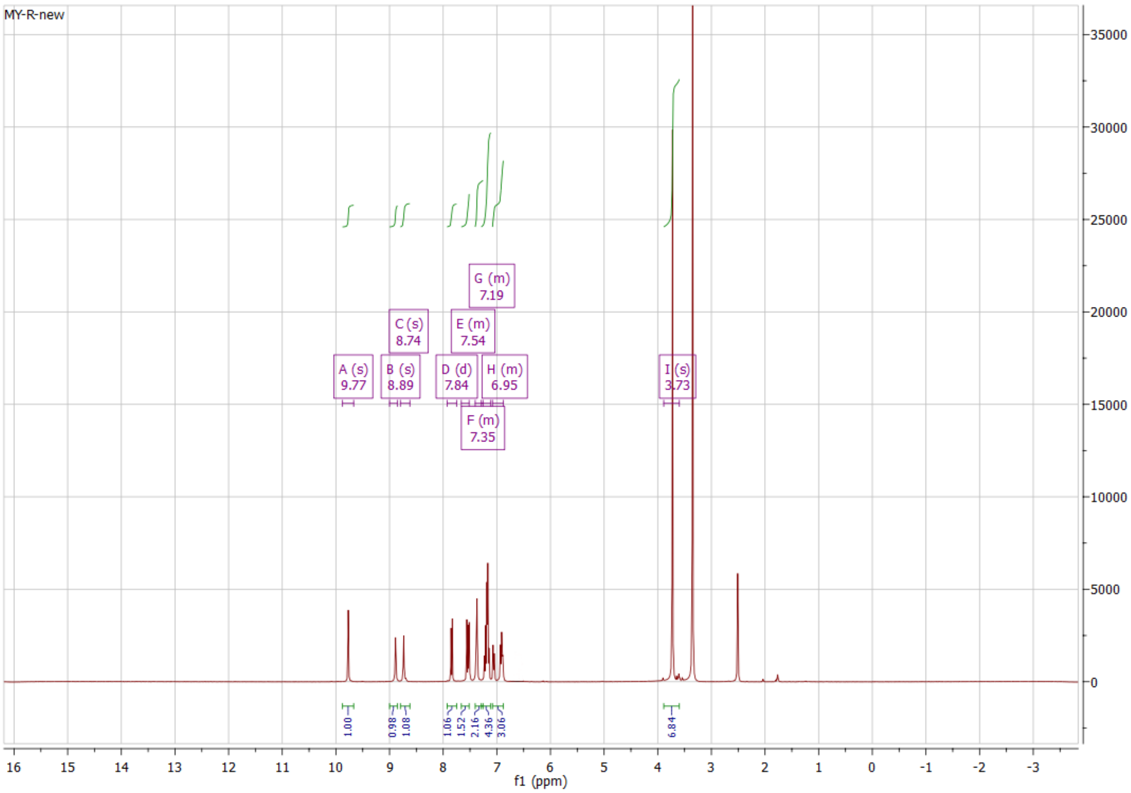

MY-R-new
A (s) 9.77
B (s) 8.89
C (s) 8.74
D (d) 7.84
E (m) 7.54
F (m) 7.35
G (m) 7.19
H (m) 6.95
I (s) 3.73
1.00
0.98
1.08
1.06
1.52
2.16
4.36
3.06
6.84
f1 (ppm)

11b

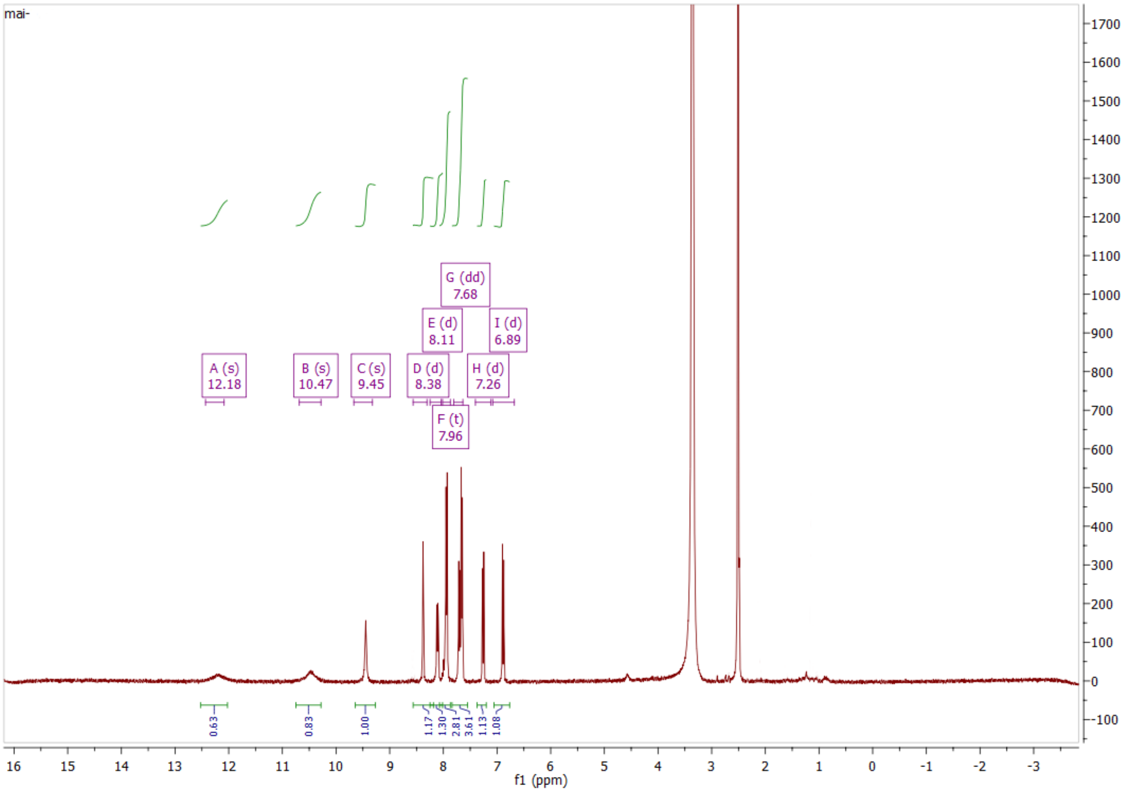

mai-
A (s) 12.18
B (s) 10.47
C (s) 9.45
D (d) 8.38
E (d) 8.11
F (t) 7.96
G (dd) 7.68
H (d) 7.26
I (d) 6.89
0.63
0.83
1.00
1.17
1.30
2.81
3.61
1.13
1.08
f1 (ppm)

11c

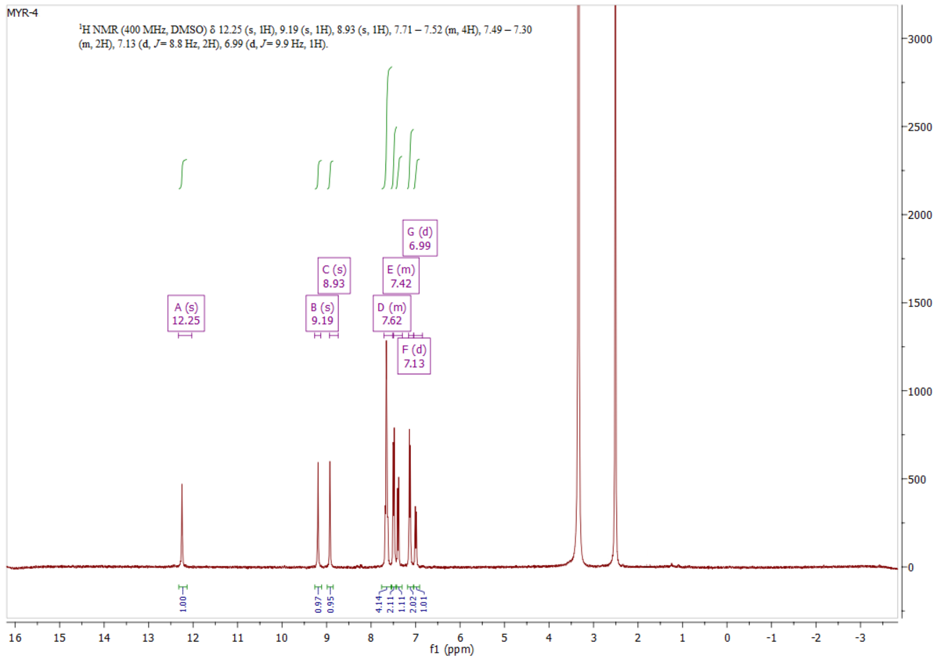

MYR-4
1H NMR (400 MHz, DMSO) δ 12.25 (s, 1H), 9.19 (s, 1H), 8.93 (s, 1H), 7.71 – 7.52 (m, 4H), 7.49 – 7.30 (m, 2H), 7.13 (d, J = 8.8 Hz, 2H), 6.99 (d, J = 9.9 Hz, 1H).
A (s) 12.25
B (s) 9.19
C (s) 8.93
D (m) 7.62
E (m) 7.42
F (d) 7.13
G (d) 6.99
1.00
0.97
0.95
4.14
2.11
1.11
2.02
1.01
f1 (ppm)

11d

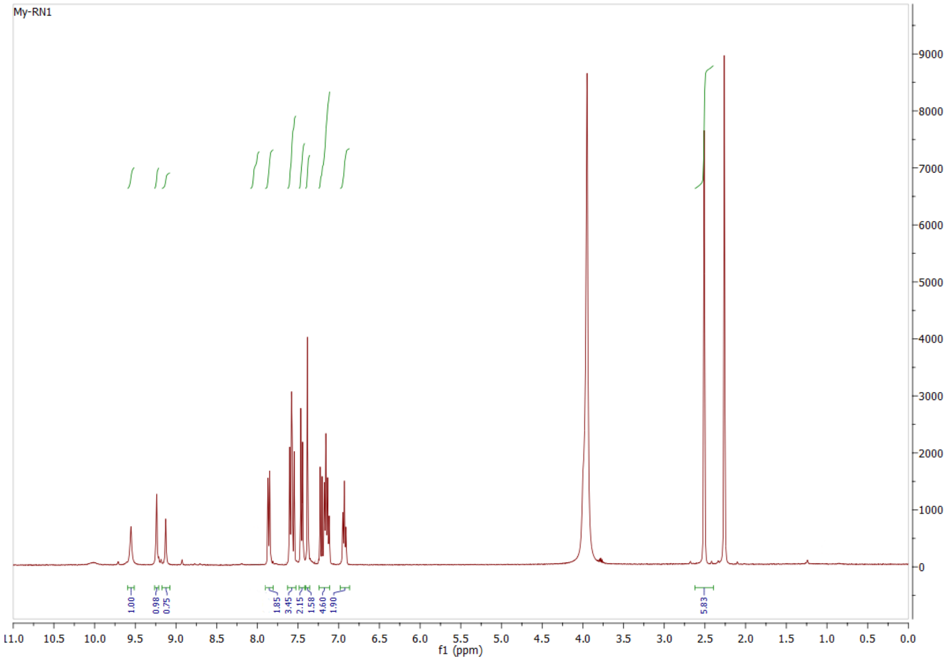

My-RN1
1.00
0.98
0.75
1.85
3.45
2.15
1.58
4.60
1.90
5.83
f1 (ppm)

11e

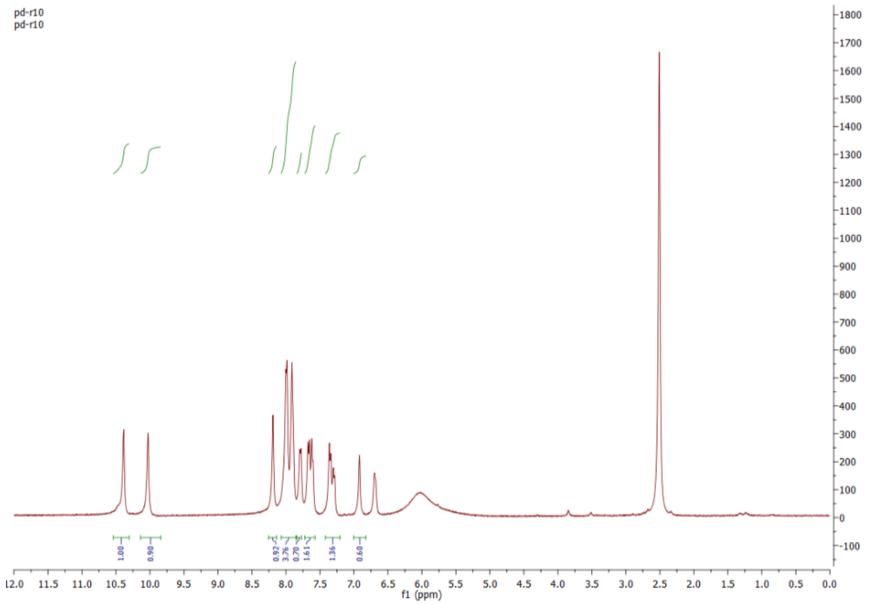
pd-r10
pd-r10
f1 (ppm)

18c

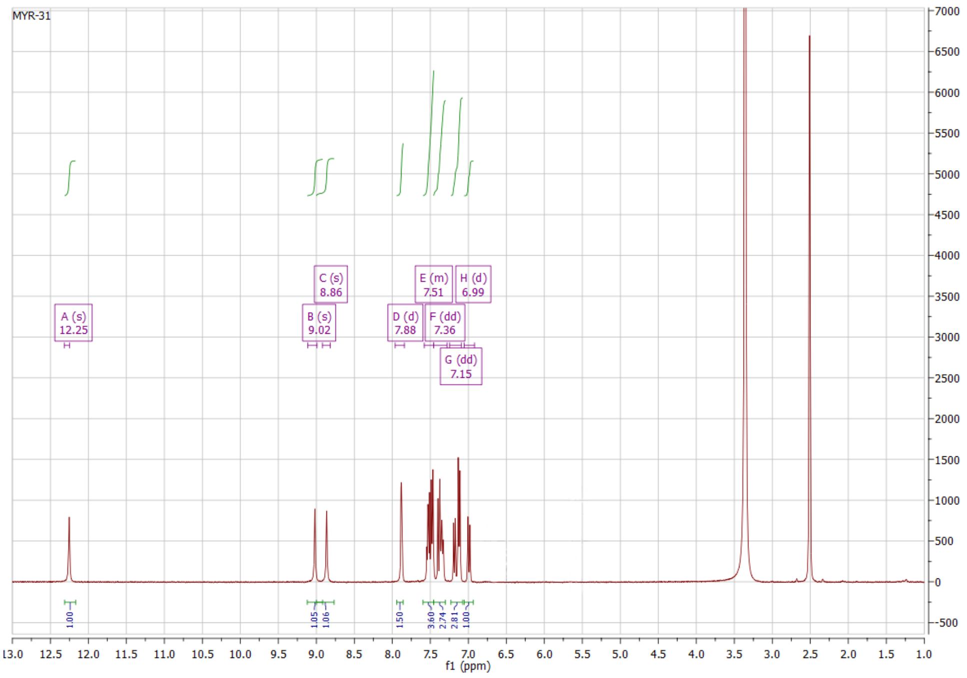
MYR-31
A (s)
12.25
B (s)
9.02
C (s)
8.86
D (d)
7.88
E (m)
7.51
F (dd)
7.36
G (dd)
7.15
H (d)
6.99
f1 (ppm)

18b

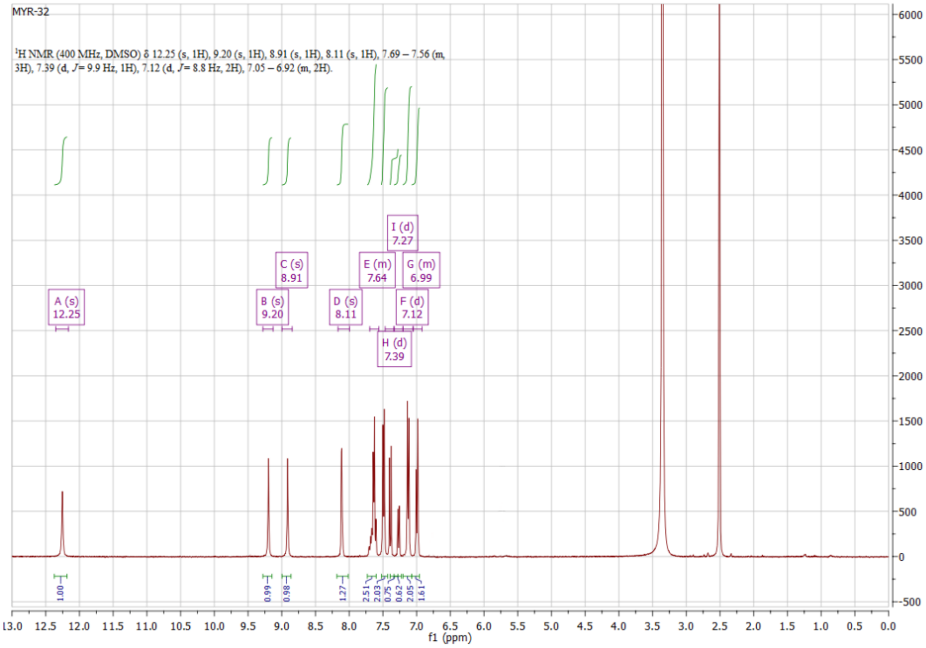

MYR-32
^{1}H NMR (400 MHz, DMSO) δ 12.25 (s, 1H), 9.20 (s, 1H), 8.91 (s, 1H), 8.11 (s, 1H), 7.69 – 7.56 (m, 3H), 7.39 (d, J = 9.9 Hz, 1H), 7.12 (d, J = 8.8 Hz, 2H), 7.05 – 6.92 (m, 2H).
A (s) 12.25
B (s) 9.20
C (s) 8.91
D (s) 8.11
E (m) 7.64
F (d) 7.12
G (m) 6.99
H (d) 7.39
I (d) 7.27
1.00
0.99
0.98
1.27
2.51
2.03
0.75
0.62
2.05
1.61
f1 (ppm)

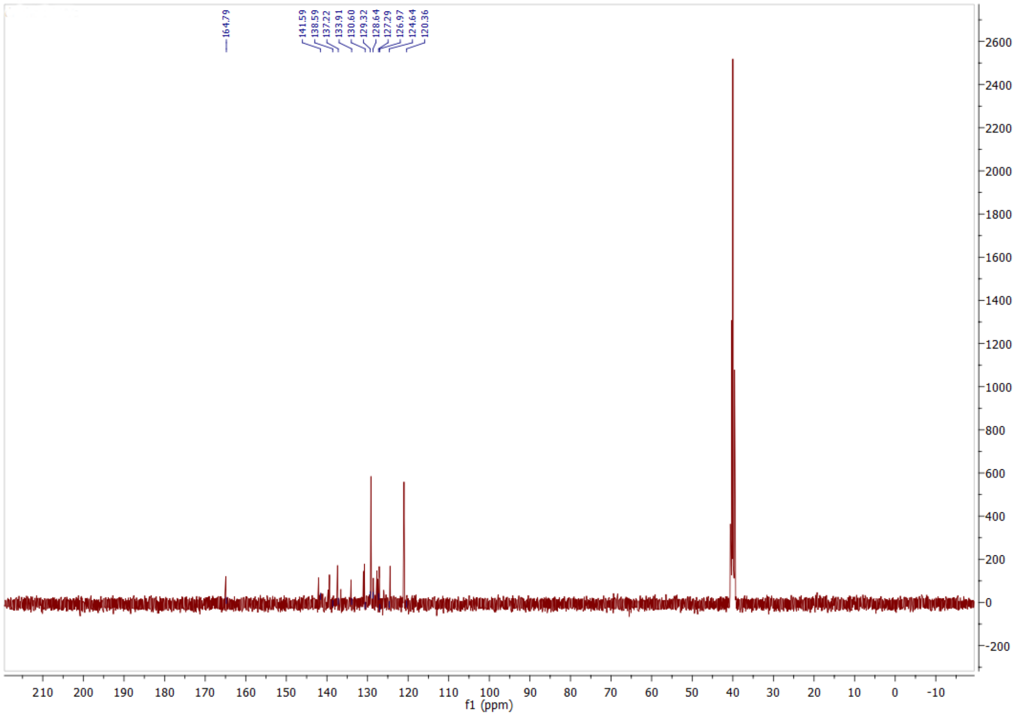

164.79
141.59
138.59
137.22
133.91
130.60
129.32
128.64
127.29
126.97
124.64
120.36
f1 (ppm)

18a

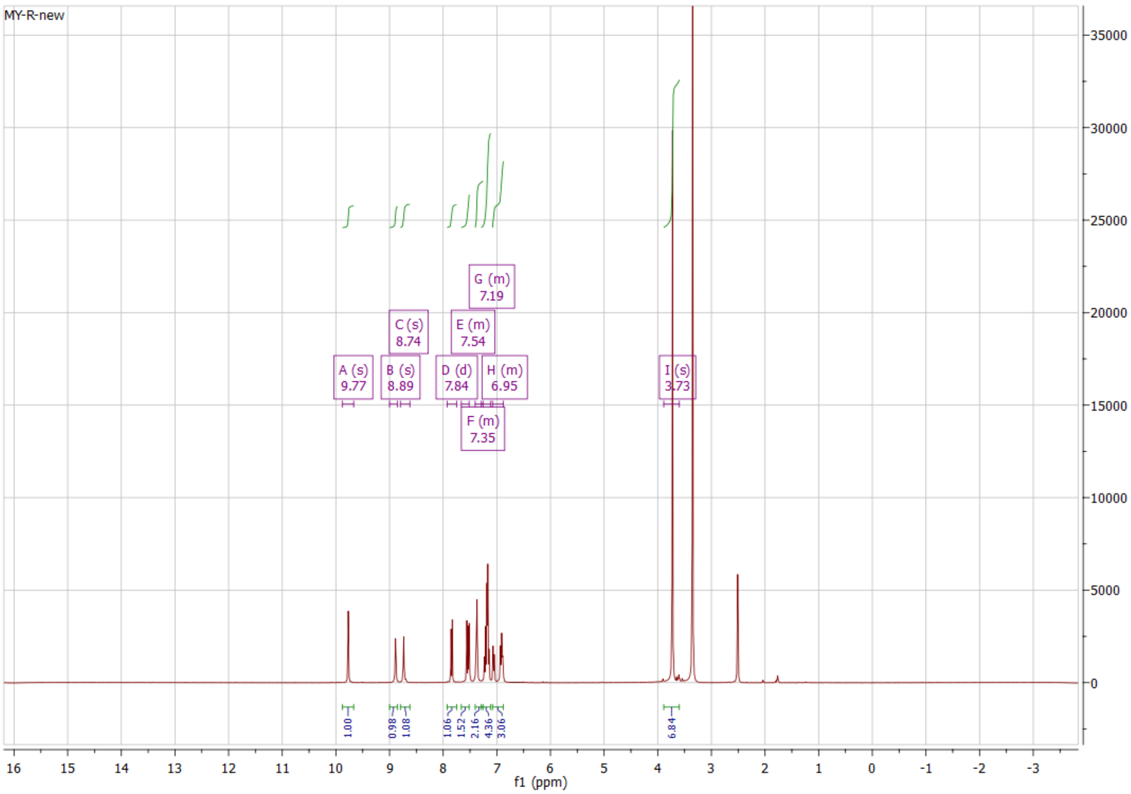
MY-R-new
A (s)
9.77
B (s)
8.89
C (s)
8.74
D (d)
7.84
E (m)
7.54
F (m)
7.35
G (m)
7.19
H (m)
6.95
I (s)
3.73
1.00
0.98
1.08
1.06
1.52
2.16
4.36
3.06
6.84
f1 (ppm)

S1 Dose response curves for measurement of VEGFR-2 inhibitory activity (IC_{50}).

S2: The relaxed potential energy surface scan of the torsional angel of the selected linker for compounds SORA, 11c, 15, 18b.

S3: Calculated (a) ligands and (b) receptors RMSD graphs for SORA, 11c and 18b over the 25ns MD trajectory.

S4: The per-residue decomposed lipophilic vdW binding energy of SORA, 11c and 18b over the 25ns MD trajectory calculated using the AMBER/MM-GBSA method in AMBER.

Fig S1:

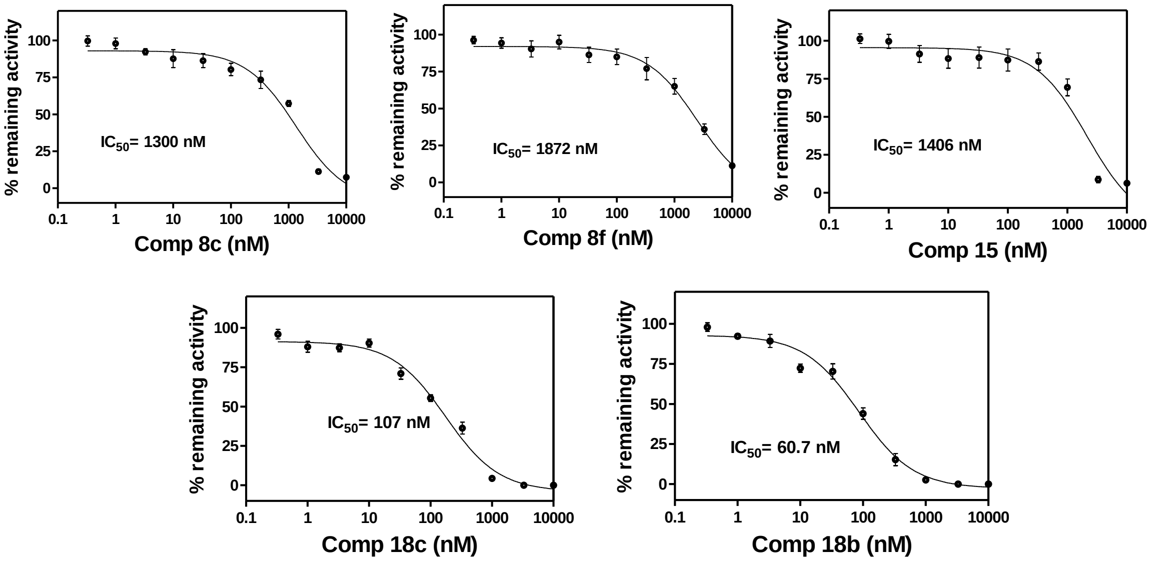

Figure S2

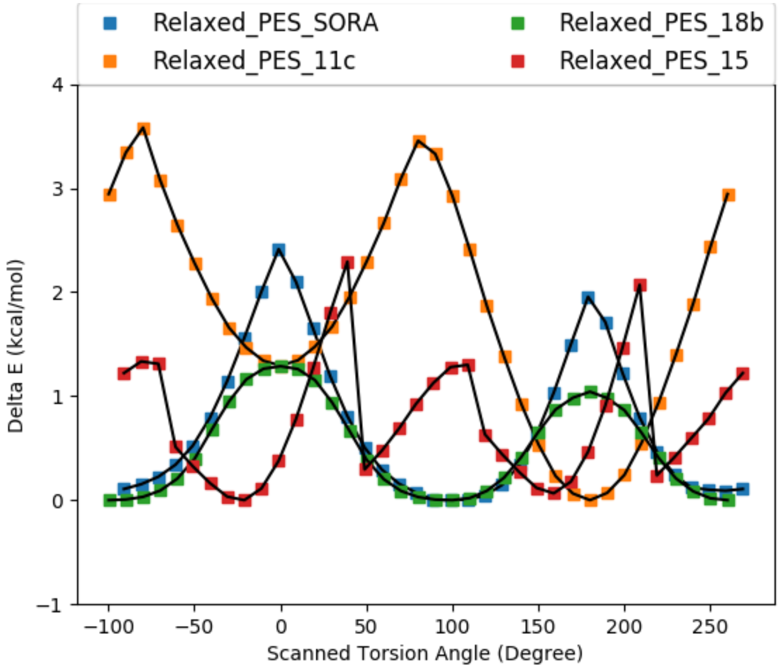

Relaxed_PES_SORA
Relaxed_PES_11c
Relaxed_PES_18b
Relaxed_PES_15
Delta E (kcal/mol)
Scanned Torsion Angle (Degree)

Figure S3:

(a)

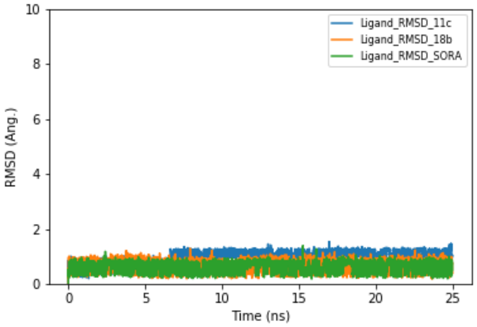

Ligand_RMSD_11c
Ligand_RMSD_18b
Ligand_RMSD_SORA
RMSD (Ang.)
Time (ns)

(b)

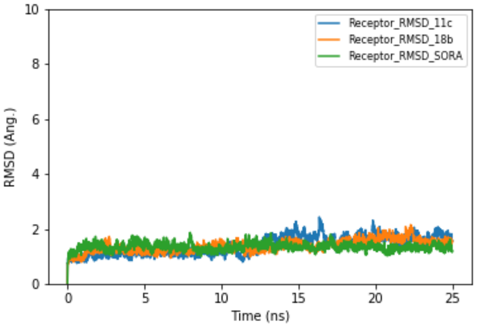

Receptor_RMSD_11c
Receptor_RMSD_18b
Receptor_RMSD_SORA
RMSD (Ang.)
Time (ns)

Figure S4

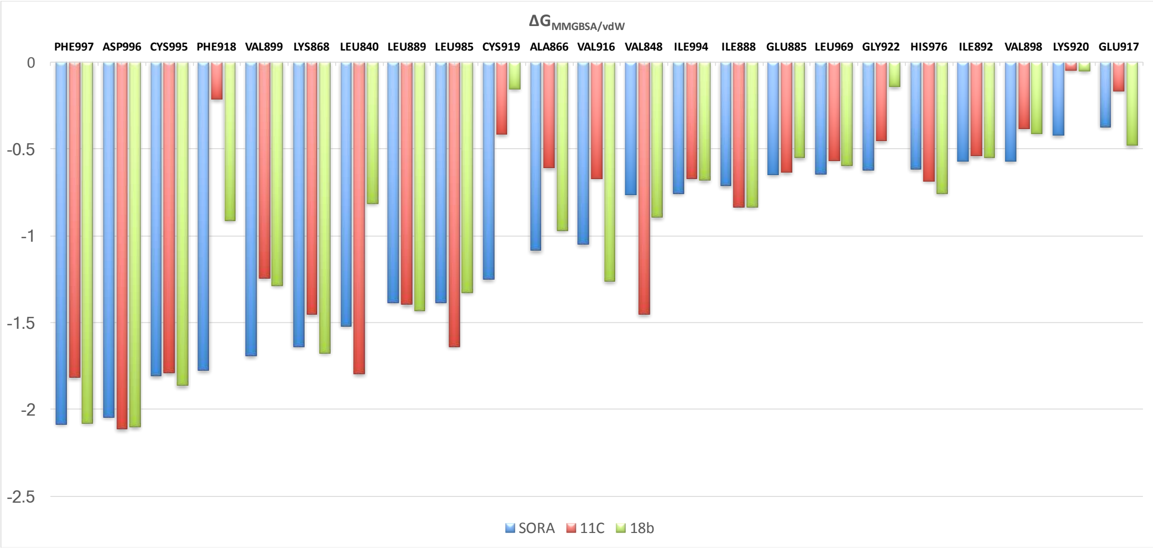

Table S1:

Selected measured and calculated activity parameters for the compounds under investigation (SORA). The AMBER/MM-GBSA scores and the H-bond occupancies are calculated over the 25ns MD simulation trajectory. Reported docking scores are for poses selected for carrying out the MD simulation. Please note that the VINA scores are reported in negative values and DLscores, NNscores and RFscores are reported in positive values.

Compound	IC50 (nm)	VINAscore	DLscore	NNscore2	RFscore	$\Delta G_{MM\text{-}GBSA}$	$\Delta G_{MM\text{-}GBSA/ELE}$	$\Delta G_{MMGBSA/vdW}$	CYS919-H-Bond Occ.	ASP996-H-Bond Occ.
**SORA**	90	-11.20	8.38	8.63	8.39	-46.87	-37.95	-59.72	86.34%	99.95%
**11C**	NA	-9.83	7.72	8.20	8.37	-36.97	-29.12	-51.84	NA	99.90%
**18B**	60.7	-10.79	7.61	8.38	8.28	-43.38	-39.98	-51.61	98.40%	99.90%
